# Converging pathomechanisms link SARS-CoV-2 infection with major neurodegenerative diseases

**DOI:** 10.1007/s10571-026-01785-9

**Published:** 2026-08-10

**Authors:** Feng Q. HeFeng, Andrea Martí-Sarrias, Heval Atas Guvenilir, Renata Baptista Vieira de Sá, Adithya Sridhar, Undine Haferkamp, Fatima El Kach-Marso, Sarah Bauermeister, Anett Hudák, Aristeidis Roubanis, Jochen Ohnmacht, Priya Sethumadhavan, Markus Ollert, Martin Hofmann-Apitius, Arcadi Navarro, Alpha Tom Kodamullil, Ole Pless, Tamas Letoha, Sandra Acosta, Rejko Krüger, on the behalf of WP4, the COMMUTE Consortium

**Affiliations:** 1 Department of Infection and Immunity, Luxembourg Institute of Health (LIH), 29 Rue Henri Koch, L-4354 Esch-Sur-Alzette, Luxembourg; 2 Barcelonaβeta Brain Research Center (BBRC), Pasqual Maragall Foundation, Carrer de Wellington, 30, 08005 Barcelona, Spain; 3 Department of Pathology and Experimental Therapeutics, Institute of Neuroscience, Medical School, Pavelló de Govern, Universitat de Barcelona, Carrer de La Feixa Llarga, s/n, 08907 Barcelona, Spain; 4 Department of Bioinformatics, Fraunhofer Institute for Algorithms and Scientific Computing (SCAI), Schloss Birlinghoven 1, 53754 Sankt Augustin, Germany; 5 OrganoVIR Labs, Emma Children’s Hospital, Department of Pediatric Infectious Diseases, Amsterdam UMC, Academic Medical Center, Amsterdam Institute for Infection and Immunity, Amsterdam Institute for Infection and Immunity, Amsterdam Institute for Reproduction and Development, University of Amsterdam, Meibergdreef 9, 1105 AZ Amsterdam, The Netherlands; 6 OrganoVIR Labs, Department of Medical Microbiology, Amsterdam UMC, Academic Medical Center, Amsterdam Institute for Infection and Immunity, University of Amsterdam, Meibergdreef 9, 1105 AZ Amsterdam, The Netherlands; 7 Emma Center for Personalized Medicine, Amsterdam University Medical Center, University of Amsterdam, Meibergdreef 9, 1105 AZ Amsterdam, The Netherlands; 8 Fraunhofer Institute for Translational Medicine and Pharmacology ITMP, Discovery Research ScreeningPort, Schnackenburgallee 114, 22525 Hamburg, Germany; 9 Department of Psychiatry, University of Oxford, Warneford Hospital, Warneford Lane, Headington, Oxford, OX3 7JX UK; 10 Pharmacoidea Ltd, Derkovits fasor 7-11, H-6726 Szeged, Hungary; 11 Transversal Translational Medicine, Luxembourg Institute of Health (LIH), 2 Rue Thomas Edison, L-1445 Strassen, Luxembourg; 12 Causality Biomodels, Kinfra Hi-Tech Park 1, Kalamassery, Cochin, 683503 Kerala India; 13 Institució Catalana de Recerca i Estudis Avançats (ICREA) and Universitat Pompeu Fabra, Doctor Aiguader, 88, 08003 Barcelona, Spain; 14 Institute of Evolutionary Biology (UPF-CSIC), Department of Medicine and Life Sciences, IBE, Universitat Pompeu Fabra, Pg Marítim de La Barceloneta, 37, 08003 Barcelona, Spain; 15 Centre for Genomic Regulation (CRG), The Barcelona Institute for Science and Technology, Doctor Aiguader, 88, 08003 Barcelona, Spain; 16 Luxembourg Centre for Systems Biomedicine (LCSB), University of Luxembourg, 6 Av. du Swing, L-4367 Belvaux, Luxembourg; 17 Parkinson Research Clinic, Centre Hospitalier de Luxembourg (CHL), 4 Rue Nicolas Ernest Barblé, L-1210 Luxembourg, Luxembourg

**Keywords:** Neurodegenerative diseases, Neurological complications, COVID, Long COVID, SARS-CoV-2 infection, Immune dysregulation

## Abstract

Neurological complications are among typical clinical manifestations, both during the acute phase of SARS-CoV-2 infection and long-term post-acute COVID-19 sequelae, i.e., Long COVID. A growing body of evidence connects SARS-CoV-2 infection to systemic inflammation, peripheral immune dysregulation, functional alteration of the blood–brain barrier (BBB), neuroinflammatory processes, mitochondrial dysfunction and pathological protein aggregation, all of which are central to the pathogenesis of major neurodegenerative diseases. In this review, we discuss the converging shared underlying molecular mechanisms that link the ‘acute’ SARS-CoV-2 infection that often only lasts for days and ‘chronic’ neurodegenerative diseases that often take decades to develop. We further review human induced pluripotent stem cells (iPSCs)-derived brain organoid and BBB models for Parkinson’s disease (PD) and Alzheimer’s disease (AD) as well as artificial intelligence (AI)-facilitated knowledge graph methods that are used to hypothesize and validate the potential mechanistic links. We propose that besides the established links between viral infections and neurodegenerative or neuroinflammatory conditions, such as Epstein-Barr virus (EBV) in multiple sclerosis or herpes zoster in AD, SARS-CoV-2 infection might be another important factor driving the future burden of the most common neurodegenerative diseases.

**Graphical Abstract:**

**Figure:**
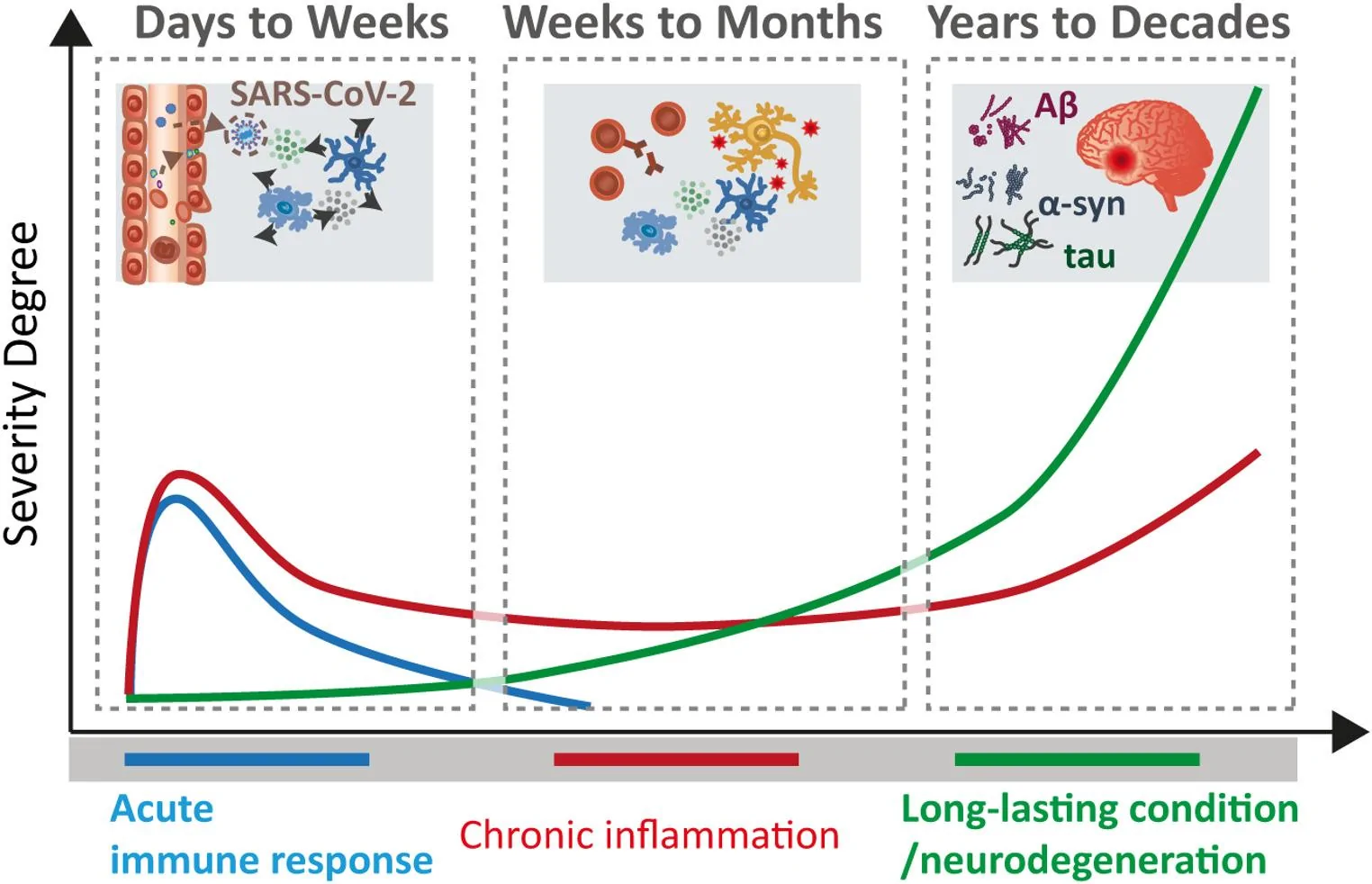
Schematic illustrating the potential link between the progression from SARS-CoV-2 infection and immune dysregulation to the development of Long COVID and eventually the subsequent onset of major neurodegenerative diseases (e.g., Parkinson's disease and Alzheimer's disease).

## Background

Neurodegenerative diseases (NDDs) encompass a heterogeneous group of disorders, including Alzheimer’s disease (AD) as the most common one and Parkinson’s disease (PD) as the fastest growing one, marked by their progressive loss of neuronal function and integrity, posing an increasing global health challenge and a major socioeconomic burden, not only due to the ever-ageing population (Jellinger 2010; Lingor et al. 2022). Evidence is accumulating to link infectious diseases to non-communicable diseases including NDDs (Netravathi et al. 2012; Wang et al. 2020; Levine et al. 2023). Historically, epidemiological studies showed a close association between the 1918 Spanish flu/influenza pandemic and encephalitis lethargica with its post-encephalitic parkinsonism (Hoffman and Vilensky 2017). More recently, viral infections, notably those caused by herpes simplex virus type 1 (HSV-1), human herpesvirus 6 (HHV-6) and Epstein-Barr virus (EBV) are being increasingly recognized as triggers to neurodegenerative processes in AD and multiple sclerosis (MS) (Rippee-Brooks et al. 2024; Duarte et al. 2023; Bjornevik et al. 2022). HSV-1 has been detected by PCR in postmortem brain tissues of people suffering from AD (Jamieson et al. 1991), and EBV-specific clones of CD8^+^ T effector memory cells have been found abundant in the cerebrospinal fluid (CSF) of AD patients, which might contribute to the neuroinflammatory process (Gate et al. 2020). In addition, a recent study using cross-sectional data from the UK Biobank couples viral encephalitis exposure with AD (Levine et al. 2023). It is worth highlighting that EBV has been unequivocally connected to the pathogenesis of MS, a neuroimmunological disease with an important neurodegenerative component. The link between EBV infection and seropositivity is now widely accepted as an important driving factor for most types of MS. The likelihood of developing MS many years after a severe primary EBV infection strongly suggests that the initial immune response to EBV plays a key role in causing MS (Lieberman 2023).

Along the same lines, the pathologies and associated morbidities of the COVID-19 pandemic causing virus, SARS-CoV-2, have also raised major concerns (Liu et al. 2020). Already in the early pandemic, it became evident that COVID-19 was not only limited to respiratory symptoms (Nalbandian et al. 2021). Among others, a wide-ranging neurological symptoms (Bonhenry et al. 2024; Wang et al. 2022a) from loss of smell and taste (Klopfenstein et al. 2020; Walker et al. 2020; Rocke et al. 2020) to more severe structural complications such as encephalitis and stroke have been reported (Nalbandian et al. 2021). Beyond complications appeared during the acute infection phase, the increased risk following SARS-CoV-2 infection to develop various long-term disorders or complications is known as Long COVID (Altmann et al. 2023; Akbarialiabad et al. 2021; Castanares-Zapatero et al. 2022; Greenhalgh et al. 2024; Crook et al. 2021), among which neurological impairments or neuropsychiatric symptoms are reported to be the most prominent post-acute sequelae over three years in a large-scale analysis (Cai et al. 2024). Neurological symptoms like memory damage and fatigue are shown to be critical to distinguish Long COVID from general pandemic-related health effects that are unrelated to a SARS-CoV-2 infection (Martins Conde et al. 2025). Moreover, SARS-CoV-2 virus has been detected in the brain and CSF of COVID-19 patients, supporting its potential neuroinvasive capacity (Stein et al. 2022). A growing body of evidence connects SARS-CoV-2 to systemic inflammation, peripheral immune dysregulation, functional alteration of the blood–brain barrier (BBB), neuroinflammatory processes, mitochondrial dysfunction and pathological protein aggregation, all of which are central to neurodegenerative pathogenesis (Lingor et al. 2022). Although it has long been thought that immune privilege occurs in the brain, the central nervous system (CNS) border tissues, such as meninges and the vasculature, especially the recently discovered meningeal lymphatic vessels (Louveau et al. 2015), have provided the anatomical basis allowing physical interactions between the CNS and peripheral immune system (Smyth and Kipnis 2025). This anatomical connectivity is particularly relevant in the context of COVID-19, as it provides potential routes through which systemic inflammation and peripheral immune cells induced by SARS-CoV-2 infection could influence brain homeostasis and potentially contribute to neurodegenerative processes. These observations further encourage researchers to explore the potential link of SARS-CoV-2 infection with NDDs in a long run (Granholm 2023). Additionally, cerebrovascular effect due to hypercoagulation and endotheliosis also regulates the integrity of the CNS, indicating comorbidities that bear implications for both the progression of major NDDs including both AD and PD (Eldholm et al. 2018; Malek et al. 2016) and the long-term effects of COVID-19 (Negrut et al. 2024). Overall, although potential links exist between COVID-19 and NDDs, those findings are primarily based on associative clinical data or epidemiological observations rather than conclusive causality evidence. This burgeoning field of study underscores the urgent need to unravel the mechanisms by which SARS-CoV-2 infection might promote or exacerbate neurodegenerative processes. As almost everyone has gotten infected by SARS-COV-2, understanding the interaction between SARS-CoV-2, including its different variants evolved over time, and future potential neurodegenerative outcomes would have a substantial impact on the socioeconomic burden in our ageing societies. Investigating this potential link may also open new avenues for mitigating the long-term global impact of SARS-CoV-2 infection through the development of novel and effective prevention strategies (Bechet et al. 2025; Bedran et al. 2024).

### COVID-19, long COVID and SARS-CoV-2 triggered processes that are implemented in neurodegeneration

**Fig. 1:**
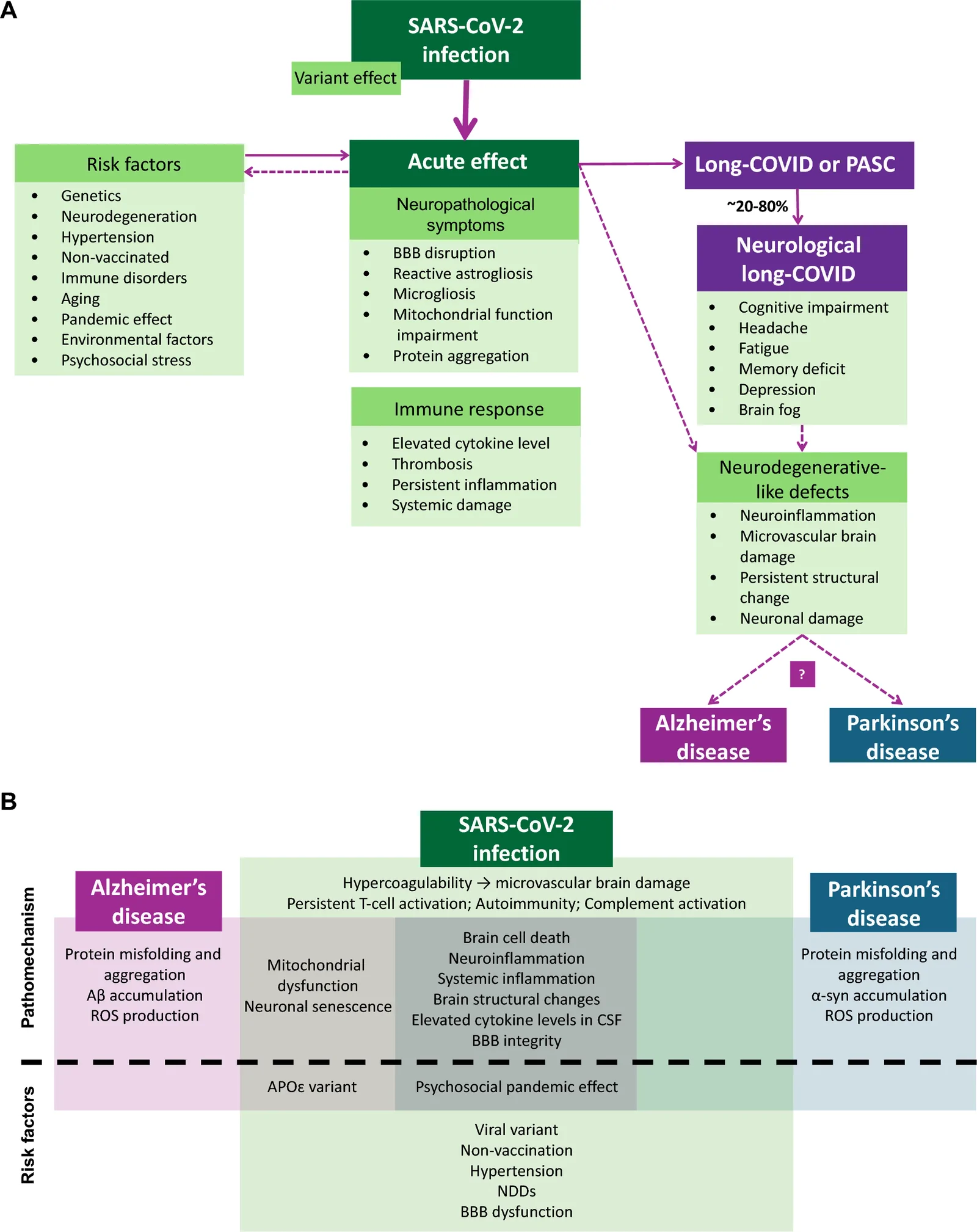
Conceptual overview of shared pathomechanisms and risk factors linking SARS-CoV-2 infection with NDDs. *a-syn* alpha-synuclein, *Aβ* beta-amyloid, *BBB* blood–brain barrier, *CSF* cerebrospinal fluid, *NDD* neurodegenerative disease, *ROS* reactive oxygen species, *PSAC* post-acute COVID-19 sequelae

Clinical data strongly support that COVID-19 can affect the CNS and has been identified as a significant factor exacerbating neurological complications, both during the acute phase of the viral infection and as part of multi-month or even multi-year long-term consequences known as post-acute COVID-19 sequelae (PACS) (Pattanaik et al. 2023; Davis 2021) or Long COVID (Altmann et al. 2023; Akbarialiabad et al. 2021; Castanares-Zapatero et al. 2022; Greenhalgh et al. 2024; Crook et al. 2021). The neurological complications in COVID-19 patients during acute infection are widespread (Singh et al. 2023), from mild symptoms like headaches, dizziness and anosmia to impaired consciousness and more severe conditions like Guillain–Barre syndrome (Liu et al. 2020), cerebellar ataxia and encephalitis (Chen et al. 2021a; Nepal et al. 2020). The impact of Long COVID on the CNS functions is clearly reflected by typical neurocognitive, psychiatric and neuropsychological impairments, e.g., brain fog, depression and anxiety (Fig. 1) (Davis 2021; Greenhalgh et al. 2022; Fischer et al. 2022). Interestingly, almost half of PD patients are suffering from those classical psychiatric symptoms (Miyoshi et al. 1996; Frisina et al. 2008). Meanwhile, the increased psychological stress experienced by patients with PD and AD during the pandemic has been shown to aggravate their symptoms and even a direct mechanistic impact cannot be excluded (Nabizadeh et al. 2022). In Long COVID individuals (Chou et al. 2021), neurological symptoms, such as brain fog, characterized by cognitive impairment, headache, fatigue, altered levels of consciousness, reduced concentration, memory and attention, have a varying prevalence from approximately 20% up to 80% depending on the cohorts of investigation (O’Mahoney et al. 2023, 2025; Davis et al. 2021), indicative of SARS-CoV-2 persistent and substantial effects on the CNS (Fig. 1). Such a high variability in prevalence might arise from a combination of various factors, such as SARS-CoV-2 variants/strains, cohort demography distribution (e.g., sex and age), pre-existing conditions, initial infection symptoms and severities, lifestyles, socioeconomic statuses/stresses and healthcare accessibility. The variability might be even related to the questionnaire design employed in the given cohort (Singh and Sagar 2021; Hirschtick et al. 2025).

The symptoms were further corroborated by histopathological analysis indicating persistent structural changes in the brain tissues of both patients recovered from an acute SARS-CoV-2 infection (Douaud et al. 2022) as well as patients suffering from Long COVID (Seo et al. 2025; Pacheco-Jaime et al. 2025). Neuropathological changes showing neuronal damage were observed in several brain regions including hippocampus, brain stem, cerebellum, thalamus, hypothalamus and cortical structures (Thakur et al. 2021). Additional evidence comes from brain imaging studies demonstrating a reduction in global brain and white matter volumes indicates a significant impact on the CNS following COVID-19, even in the absence of long-term observable symptoms (Douaud et al. 2022), similar to what has been reported in AD patients (Chouliaras and O’Brien 2023). Several functional brain imaging studies provide evidence showing a SARS-CoV-2 infection is linked with triggering PD pathology in DAT-SPECT scans (Cohen et al. 2020) or associated with functional connectivity changes in Long COVID individuals, as evidenced by functional magnetic resonance imaging (fMRI) (Carreras-Vidal et al. 2025). However, the pathogenic mechanisms how mild SARS-CoV-2 infection or severe COVID-19 lead to neurological symptoms and damages still largely remain elusive.

### Neurotropism of SARS-CoV-2

SARS-CoV-2 infection can result in neurological changes either through direct invasion of specific neurons or resident immune cells in the CNS, or indirectly via systemic effects that also impact neurological functions. Infection of neural tissues by the virus can be a straightforward explanation of neurological consequences following the respiratory tract infection. Viral RNA can be detected in brainstem of infected individuals (Emmi et al. 2023). The angiotensin-converting enzyme 2 (ACE2) receptor, to which the SARS-CoV-2 spike protein (S) binds, is widely expressed in various body cells (Li et al. 2020; Hikmet et al. 2020) including those in respiratory tract (Zou et al. 2020); hence by directly infecting the nasal cavity, SARS-CoV-2 can enter the brain through olfactory sensory neurons (Jiao et al. 2021; Meinhardt et al. 2021), a similar scenario to that of ‘brain first’ PD (Horsager and Borghammer 2024). Additionally, trans-synaptic transfer has been hypothesized as a potential route where the virus could spread from neuron to neuron, further facilitating the virus’ CNS invasion (Lima et al. 2020). Infection of astrocytes and pericytes could also indirectly facilitate viral neuroinvasion by damaging the BBB, especially aged associated BBB (Adesse et al. 2022). Within the brain, the detection of viral proteins and RNA (including subgenomic RNA), within multiple cell types (microglia, astrocytes and neurons) in brain tissue points towards local viral replication (Hanley et al. 2020; Martin-Villamil et al. 2022; Costa et al. 2022; Cosentino et al. 2021; Ding and Zhao 2023). This is further supported by obtaining infectious viral particles from postmortem brain tissue (Stein et al. 2022). Notably, low levels of SARS-CoV-2 transcripts have been detected in the neurons of substantia nigra tissue donated from COVID-19 patients, further indicative of a potential link to the development of PD (Yang et al. 2024; Emmi et al. 2023).

Meanwhile, it has also been proposed that SARS-CoV-2 presence in the brain could be a stochastic process that is incidental rather than mechanistically causative for neuropathological manifestation. This is supported by case reports where substantial brain pathology is observed despite the minimal presence of viral RNA (Poloni et al. 2022). In the meantime, viral RNA and proteins in the CNS appear to differ between patients with and without neurological symptoms (Cosentino et al. 2021). Another study also indicates that the neuropathology following COVID-19 may not be related to direct viral infection of the cells in the CNS, but via BBB alteration (Song et al. 2023). Regardless of viral presence in the brain, pathological hallmarks of COVID-19 in the CNS include neuronal damage and neuroinflammation-associated pathology such as encephalitis, microgliosis, reactive astrocytosis, inflammatory infiltrates (Cosentino et al. 2021). Different works have also shown more indirect lesions related to cerebrovascular damage including hypoxia-ischemic lesions, hemorrhagic lesions and microthrombi and small vessel diseases in patients with and without neurological impairment, as nicely summarized elsewhere (Cosentino et al. 2021). These indirect lesions may arise from an exacerbated immune response following respiratory infection, along with cerebrovascular injury and hypoxic and metabolic alterations, as reported in COVID-19 patients.

### SARS-CoV-2 triggered systemic inflammation and immune dysregulations

Acute SARS-CoV-2 infection could lead to a cytokine storm, a severe hyperinflammatory state marked by a substantial increase of cytokines, which can cause extensive damage to both the central and peripheral nervous systems (Thepmankorn et al. 2021). This cytokine profile is similar to that observed in secondary hemophagocytic lymphohistiocytosis, a life-threatening condition characterized by extreme hypercytokinemia and multi-organ failure (Thepmankorn et al. 2021; Meng et al. 2021). While attempting to control viral infection, the immune system's response can inadvertently lead to neurological complications such as headaches and impaired vigilance, which occur in up to 12% of COVID-19 patients experiencing a cytokine storm (Thepmankorn et al. 2021; Pergolizzi et al. 2022).

**Fig. 2:**
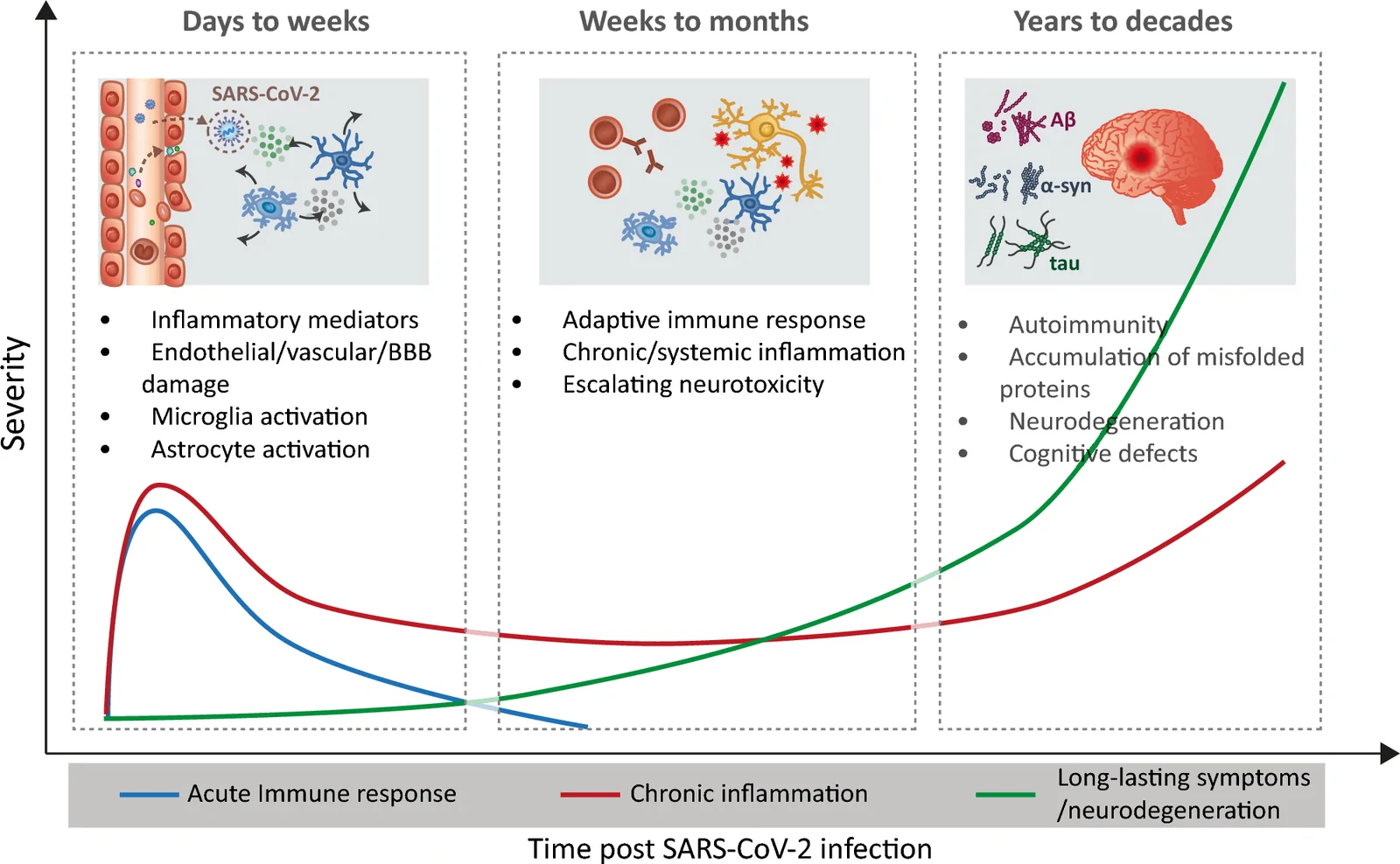
Proposed timeline and framework from SARS-CoV-2 infection, neuroimmune response, BBB compromise, immune dysregulation to the development of neurological symptoms. *BBB* blood–brain barrier

Systemic inflammation triggered by SARS-CoV-2 infection also profoundly impacts neurological functions via structural changes related to inflammation-induced infiltration of immune cells into the brain and mediators leading to structural and functional changes (Greene et al. 2024). Following SARS-CoV-2 infection, inflammatory responses such as vasculitis, glial activation, and upregulated inflammatory factors are commonly observed (Zhang et al. 2021) (Fig. 2). Recently, various cytokines were observed increased also in CSF of acute COVID with neurological manifestations (Freitas et al. 2025), suggesting a more direct impact on CNS. Furthermore, neuropsychiatric syndromes, such as delirium (Sankowski et al. 2015), can be directly induced by soluble mediators such as tumor necrosis factor (TNF), interleukin (IL)-1 or High Mobility Group Box 1 (HMGB1) or indirectly by activating microglia and astrocytes. Elevated serum levels of proinflammatory markers such as IL-6 and soluble TNF receptor (sTNFR1) as well as anti-inflammatory cytokine IL-10 have been directly associated with poor outcomes and neurological sequelae in cases of COVID-19-related encephalopathy (Tutal Gursoy et al. 2023). The release of inflammatory cytokines can trigger the coagulation cascade and suppress anticoagulant pathways, making patients prone to both thrombotic and hemorrhagic cerebrovascular events (Bhaskar et al. 2020) and significantly increasing the risk of cerebrovascular accidents, such as strokes, in COVID-19 patients (Thepmankorn et al. 2021). Encephalopathy, a severe general brain dysfunction, can arise from cytokine storms, with increased cytokine levels causing metabolic dysregulation and excitotoxicity. This has been particularly noted in patients with influenza virus-associated encephalitis/encephalopathy, where elevated TNF-α levels were observed in both serum and CSF (Shukla et al. 2021; Barbosa-Silva et al. 2021; Tutal Gursoy et al. 2023). The persistent replication of SARS-CoV-2 and the syncytia formation from infected cells can stimulate cytokine and autoantibody (auto-Ab) production, leading to long-term effects on brain function including memory (Ding and Zhao 2023). The induction of a cytokine storm in systemic circulation can trigger neuroinflammatory cascade reactions, which can subsequently have an impact on the BBB and activate microglia- and astrocyte-borne Toll-like receptors (TLRs), thereby leading to neuronal tissue damage (Kumar et al. 2021) (Fig. 2). Thus, a cytokine storm can lead to significant neurological impairments and highlight the interconnectedness of systemic inflammation and neurodegenerative processes in the long run.

In the meantime, viral persistence, as evidenced by the detection of SARS-CoV-2 reservoirs in diverse tissues, as well as viral RNA or circulating viral S protein antigens, has been well documented across multiple independent works, both in original research articles (Zuo et al. 2024; Swank et al. 2024) and in comprehensive reviews (Proal et al. 2023; Prakash et al. 2025). In a multicohort study involving 706 individuals, despite variability in S protein antigen positivity rates across cohorts, Long COVID was associated with an approximately twofold increase in average in the likelihood of detecting circulating S protein antigens, with an overall positivity rate of ~ 43% in affected individuals compared with asymptomatic controls (Swank et al. 2024). Notably, evidence of persistent viral material extends to tissue compartments: intracellular SARS-CoV-2 single-stranded RNA encoding the S protein was detected in rectosigmoid lamina propria samples from all five examined patients with Long COVID, even two years after the initial infection (Peluso et al. 2024). Although the development of NDDs may take decades, persistent SARS-CoV-2 viral presence over a two-year period could already be sufficient to drive chronic systemic inflammatory cascades, as discussed in the following sections, thereby contributing to prolonged pathophysiological changes and the emergence of neurological manifestations.

Together, these various works, although including many reviews and preliminary analyses, highlight the critical need for a deeper understanding into the interaction between viral infections, systemic inflammation and neuroinflammatory mechanisms to develop targeted therapies that can mitigate related severe effects.

### Long COVID associated immune dysregulations

Although the precise molecular mechanisms underlying the development of Long COVID still remain largely unknown (Crook et al. 2021; Davis et al. 2023), several potential mechanisms have been proposed (Fig. 1). These include persistent immune system dysregulation leading to chronic inflammation and potential tissue damage, neuroinflammation, neurodegeneration and demyelination. Recently, a correlation has been shown between persistent complement dysregulations or spontaneous persistent T-cell responses and Long COVID from those initially infected with early B.1 lineage (D614G, also known as Clade G or European variant) (Cervia-Hasler et al. 2024; Krishna et al. 2024) (Fig. 1). These findings all suggest that persistent immune activations, although in different immune arms including both adaptive and innate immunity, may contribute to the development of Long COVID. These reports indicate that SARS-CoV-2 infection might trigger immune dysregulations resulting in the development of Long COVID that might eventually lead to long-term neurological manifestations (e.g., PD and AD) (Fig. 2).

In a US California cohort (n = 27 Long COVID individuals vs n = 16 fully recovered people), individuals, initially infected with wildtype strain, presenting persistent Long COVID symptoms for more than three months showed increased circulating levels of several chemokines (e.g., CCL21, CCL22 and CXCL10) and cytokines (e.g., also including TNF-α) eight months post infection compared to recovered individuals (Yin et al. 2024). The observation is evidently linked to Long COVID as the examined samples were taken before the occurrence of vaccination or re-infection. In parallel, in a German population-based cohort with many more participants (initially recruited for a digital health study), although with less stringent inclusion criteria (more than 80% of participants with at least one dose of the COVID-19 vaccine), the authors found that plasma levels of TNF-α, IL-1β and IL-6 were increased in individuals, who were initially infected mainly with early B.1 lineage (D614G) or Alpha, suffering from PASC for more than eight months (Schultheiß et al. 2022). Another UK study comprehensively analyzed 368 plasma proteins in 657 participants (426 out of 657 participants had developed at least one Long COVID symptom) between three and eight months following initial COVID hospitalization and even identified several subgroups of Long COVID, each of which showed an enhancement of different inflammatory markers (Liew et al. 2024). Recruited patients were all hospitalized during acute infection mainly with early B.1 lineage (D614G) between Feb 2020 and Jan 2021. As Long COVID is such a heterogeneous condition and shows varying magnifications or varying combinations of symptoms among different individuals (Thaweethai et al. 2025), more detailed analyses are still required to investigate symptom-specific immune dysregulations.

In addition to the observed systemic inflammation, Long COVID individuals also preferentially expressed several exhaustion markers (e.g., PD-1 and CTLA4) in CD8 T cells, which was independently confirmed by several studies based on those initially infected either with early B.1 lineage (D614G) or Alpha (Aid et al. 2025) or with a wide range of variants including B.1 lineage (D614G), Alpha, Delta and Omicron (Gao et al. 2025). Those works from several independent groups together strongly indicate that chronic and recurrent immune stimulation (Fig. 1B) might be associated with Long COVID symptoms, although causal evidence that T-cell exhaustion causes long COVID is still missing. The observed exhaustion pathways coexisted with the upregulation of JAK-STAT and IL-6 pathways for > 180 days in the Long COVID group relative to the convalescent controls (Gao et al. 2025). That work together with several others (Phetsouphanh et al. 2022; Yin et al. 2024) suggest that IL-6 and JAK-STAT pathways might be important for the development of Long COVID symptoms.

Furthermore, individuals with Long COVID exhibited discoordinated humoral and cellular immunity (Yin et al. 2024). In the meantime, the coordinated immune response between adaptive and innate immune arms was confined only to initially mild individuals during primary infection, but not in hospitalized COVID patients during the first days of acute infections (Capelle et al. 2022). Coordinated immunity seems to be crucial to a proper response during both acute infection phase and long-term recovery stage.

However, the UK-Sweden joint study (the first cohort with n = 70 Long COVID individuals initially infected with a wide range of variants including B.1 lineage (D614G), Alpha, Delta and Omicron vs n = 70 healthy convalescent controls recruited in UK; the second cohort with n = 95 Long COVID individuals vs n = 30 controls in Sweden) was unable to identify a clear association between immune cell lineage composition and antiviral T-cell immunity with Long COVID symptoms (Gao et al. 2025), indicating a necessary deeper investigation in this direction. The major difference between the US California and UK-Sweden cohorts is that the UK-Sweden cohorts were recruited between Mar and Oct 2022 from those initially infected with a wide range of variants including B.1 lineage (D614G), Alpha, Delta and Omicron over a long period between Mar 2020 and Oct 2022 while the US California cohort was sampled between Sep 2020 and Apr 2021 mainly from those initially infected with wildtype strain, which might indicate variant-specific responses (Hedberg and Naucler 2024; Magnusson et al. 2022) (Fig. 1A), although vaccine protection also exists after vaccination rollout (Caspersen et al. 2025; Rong et al. 2024).

Meanwhile, as a much higher portion of females than males are experiencing Long COVID (Shah et al. 2025; Martins Conde et al. 2025), sex-specific immunological responses (Sharma et al. 2025; Altfeld et al. 2026) might exist. In fact, such responses already occur even in the acute infection phase. For instance, with initial moderate symptoms, males show higher innate cytokines (e.g., IL-8, IL-18) and expanded non-classical monocytes, whereas females exhibit stronger T-cell activation; correspondingly, poor T-cell responses predict worse outcomes in males, while elevated innate cytokines predict progression in females (Takahashi et al. 2020). With severe COVID disease, males display higher IL-6, IL-8, and monocyte chemoattractant protein-1 (MCP-1) levels and increased neutrophils and monocytes, while females have greater B-cell proportions (Qi et al. 2021). Females also mount stronger and earlier IgG responses, including RBD-specific antibodies (Abs) (Huang et al. 2021a). Notably, neutralizing autoantibodies (auto-Abs) against type I IFNs occur more frequently in males and contribute substantially to life-threatening diseases (Bastard et al. 2020). At the molecular level, male patients show increased co-expression of ACE2 with viral entry factors and higher TLR7 expression, suggesting enhanced viral entry and innate activation (Hou et al. 2021). Collectively, females exhibit more robust adaptive immunity, whereas males are prone to dysregulated innate responses and impaired IFN signaling during the acute phase, patterns that may differentially influence disease progression and Long COVID related manifestations in a long run.

As most of autoimmune diseases are much more prevalent in females than males (Cooper and Stroehla 2003; Pisetsky 2023; Quintero et al. 2012), female-biased observations often indicate the existence of autoimmune responses. Therefore, autoimmunity has also been speculated to also play a crucial role (Fig. 1B), with some proposing that auto-Abs might target not just the respiratory and cardiovascular systems but also the nervous system (Prüss 2021). Several studies have reported or summarized auto-Abs in COVID-19 patients (Lee et al. 2023; Wang et al. 2021b) or have shown certain auto-Abs are associated with Long COVID symptoms based on those initially infected with B.1 lineage (D614G), Alpha or Delta (Son et al. 2023). Moreover, the neurotropism nature of SARS-CoV-2 could trigger an autoimmune response, complicating the neurological manifestations associated with the virus (Bedran et al. 2024). However, in the German population-based cohort with those initially infected mainly with early B.1 lineage (D614G) or Alpha (Schultheiß et al. 2022), Long COVID outcomes were not associated with the tested auto-Abs against (e.g., rheumatoid factor, antinuclear Abs and anti-phospholipid Abs), guaranteeing further investigation in this regard.

In the meantime, the inconsistency was noticed between different cohorts, possibly owing to varying inclusion/exclusion criteria, variable recruitment periods with distinct dominant variants and participant demographics. For instance, the anti-SARS-CoV-2 Ab response (i.e., humoral response) was reported elevated in Long COVID individuals, who were initially mainly infected with the WA-1 wildtype strain, in a study led by the Yale-Mount Sinai groups (Klein et al. 2023), while a suboptimal neutralization Ab response was observed among Long COVID individuals, who were initially infected with a wide range of variants including B.1 lineage (D614G), Alpha, Delta and Omicron, in the UK-Sweden cohorts (Gao et al. 2025). Not to mention different cohorts, even within the same US California cohort an opposite change between IL-4 and IL-5 was observed, although both being classical Th2 cytokines, in individuals with Long COVID, initially mainly infected with wildtype strain, relative to the recovered persons (Yin et al. 2024). All these various facts highlighted the necessity to further deeply investigate the immunological link between COVID, Long COVID and long-term neurological complications.

### SARS-CoV-2 triggers structural and functional alterations of the vasculature and the BBB

Brain capillaries are lined in their circumference by specialized endothelial cells, termed brain capillary endothelial cells (BCECs). They represent the main component of the BBB and interact physically and functionally with pericytes, astrocytes, neurons, microglia and extracellular matrix components in a highly organized manner. As a hallmark, BCECs are covered by specialized astrocyte end feet and they show an increased expression of tight junctional proteins, solute carriers and efflux transporters when compared to peripheral vasculature (Keller 2013; Hawkins and Davis 2005). Functionally, the BBB fulfills physical-, metabolic- and transport-barrier functions and regulates biochemical and cellular traffic, thereby crucially maintaining homeostasis of the brain (Abbott and Friedman 2012; Neuhaus and Noe 2009). Notably, microvascular damage, and specifically dysfunction of the BBB, are common features of aging (Knox et al. 2022) and the diseases most-associated with coronavirus infection severity and neurological symptoms (e.g., cerebrovascular disease, diabetes mellitus and hypertension). Structural and functional alterations of the BBB can be found in numerous brain disorders, such as stroke, epilepsy, schizophrenia, brain trauma and MS, also including several NDDs, e.g., Huntington´s disease, amyotrophic lateral sclerosis, PD and AD (Neuwelt et al. 2011; Greene et al. 2018; Lau et al. 2024). In addition, BBB breakdown also leads to the increased uptake of inflammatory mediators like cytokines, chemokines, peripheral leukocytes and CD4 + T cells into brain parenchyma thereby accelerating disease progression. Red blood cell extravasation as well as infiltration by peripheral macrophages and neutrophils reported in AD postmortem studies further suggest innate immune system activation in the brain contributing to the pathophysiological changes (Sweeney et al. 2019; Nishihara et al. 2020; Engelhardt et al. 2017). BBB leakage has also been intensively reported in PD patients as summarized elsewhere (Lau et al. 2024). These data already suggest that different diseases might share common mechanisms affecting BBB functionality. However, many mechanisms underlying the onset, progression and severity of CNS diseases are not completely understood, in particular with regard to BBB functionality (Profaci et al. 2020).

The main SARS-CoV-2 entry receptor, ACE2, is broadly expressed also in blood vessel endothelium (Hikmet et al. 2020). Multiple downstream effects of SARS-CoV-2 have been reported resulting from ACE2 engagement. These include vascular dysfunction and endothelial damage in multiple tissues, including the brain. This damage can even be exacerbated with pre-existing conditions, e.g., older age, cardiovascular disease, hypertension or diabetes. Alteration of cerebral blood flow and microvascular damage can result in hypoxia and in turn neuronal injury, initiating or progressing the development of NDDs (MacLean et al. 2020). Besides a direct neuroaxonal CNS invasion via olfactory, trigeminal, or vagus nerves, SARS-CoV-2 may directly infect the BBB, thereby disturbing its structural, molecular and functional integrity. As an alternative CNS entry route, SARS-CoV-2 can infect circulating immune cells (e.g. monocytes) in the periphery which may then cross the BBB, a process which is facilitated by proinflammatory cytokines and chemokines (Bell et al. 1996). Passage of infected monocytes across the BBB may occur via transcellular pathways involving intercellular adhesion molecule 1 (ICAM-1, CD54)-mediated transport, which is upregulated by proinflammatory TNF-α. Alternatively, paracellular leakage may occur due to increased matrix metalloproteinase 9 (MMP-9) enzyme activity (upregulated by TNF-α) and subsequent degradation of tight junction and the basement membrane (Rempe et al. 2016). Monocytes, T cells and dendritic cells all express and release MMP-9 (Rempe et al. 2016).

In addition to compromising BBB integrity (Fig. 1B), MMPs activate neuroinflammatory pathways and provide signals for further production and secretion of pro-inflammatory cytokines (Rempe et al. 2016). Furthermore, infected macrophages or microglia produce chemokines recruiting more infected leukocytes, resulting in pro-inflammatory cytokines (e.g. IL-6, IL-1β, TNF-α) with neurotoxic potential (Desforges et al. 2014). Microvascular dysfunction may also occur following direct viral binding of ACE2 expressed on the capillary endothelium of the BBB, triggering an inflammatory cascade and release of many different proinflammatory cytokines have neurotoxic potential (Alquisiras-Burgos et al. 2021; Chen et al. 2022; Greene et al. 2024). Furthermore, proinflammatory cytokines and chemokines [e.g. macrophage inflammatory protein 2 (MIP-2), IL-8, IL-6 and MCP-1)] increase BBB permeability and facilitate monocyte and leukocyte transmigration across the BBB, propagating damaging neuroinflammatory pathways.

A compromised BBB in turn allows more inflammatory cytokines, immune cells, and possibly neurotoxic substances to enter the brain, further destabilizing neuronal environments and promoting pathological protein aggregation (for details, refer to the next section) (Takata et al. 2021) in a positive feedback loop. Severe cases of COVID-19 are often associated with systemic inflammation, including the release of cytokines and chemokines that can disrupt the tight junctions of the BBB (Greene et al. 2024). Patients with SARS-CoV-2 infection develop a systemic pro-inflammatory cytokine response [i.e., increased levels of IL-1β, IL-2, IL-6, IL-7, IL-12, granulocyte colony-stimulating factor (G-CSF), interferon (IFN)-γ, IP-10, MCP-1, MIP-1α, TNF-α and vascular endothelial growth factor (VEGF)], which likely contributes to BBB dysfunction (Huang et al. 2021b; Shlosberg et al. 2010; Greene et al. 2024; Becher et al. 2017). In summary, SARS-CoV-2 patients with pre-existing BBB dysfunction are likely to be at higher risk of CNS invasion of SARS-CoV-2. Together, these findings suggest that damage to the BBB, either due to a pre-existing comorbidity or virus-induced pro-inflammatory response, may facilitate the extravasation of infected immune cells from the bloodstream to the brain, which might play a crucial role in the onset or progression of NDDs.

There is evidence for a well-established relationship between the accumulation of vascular white matter lesions in the brain and cognitive decline (Román 2002; Stricker and Kiprov 1993; Gorelick et al. 2011), beyond defective BBB permeabilization. For instance, cerebral amyloid angiopathy (CAA), a severe and fast progressive dementia, is driven by the accumulation of beta-amyloid (Aβ) plaques adjacent to microvessels generating locally contained bleedings. Cerebral parenchymatous bleeding induces neuronal death and neurodegeneration. Obviously, CAA is closely intersecting with pathways involved in AD, as both pathologies are driven by impaired Aβ clearance, leading to increased vascular Aβ causing an overload of perivascular clearance mechanisms (Greenberg et al. 2020). Furthermore, the overlap between vascular and AD pathology was frequently reported (Iadecola and Anrather 2025). In the meantime, SARS-CoV-2 was shown to bind to ACE-2 expressing endothelial cells and infect them, leading to direct neuroinvasion (Krasemann et al. 2022). Moreover, COVID-19 also enhances clotting pathways and hypercoagulability (Abou-Ismail et al. 2020), which can lead to microvascular brain damage and even territorial strokes in severe cases. Reports have also shown that COVID-19 association with thrombotic and other hemorrhagic conditions could also exacerbate microvascular complications (Gąsecka et al. 2021; Roberts et al. 2020) and might eventually accelerate the development of NDDs in the long run.

There is a growing body of human data independent of in vitro and postmortem evidence for the presence of SARS-CoV-2 in the brain. A multicenter study of n = 127 COVID-19 patients (Jarius et al. 2022) revealed that the most frequent pathological finding in CSF was blood-CSF barrier (BCB) dysfunction, occurring in 50% of samples and often persisting for several weeks. Despite these barrier issues, the study found a striking absence of intrathecal inflammation with only 11% of samples showing increased white cell counts and negativity of SARS-CoV-2 CSF PCR in 76 tested cases with neurological symptoms. Findings instead showed high rates of albuminocytological dissociation and frequently elevated levels of proinflammatory cytokines such as IL-6 and IL-8, which often remained positive for months in both serum and CSF. Additionally, routine anti-neuronal and anti-glial auto-Abs were mostly absent, further suggesting that neurological complications are likely driven by systemic inflammation or cerebrospinal endotheliopathy rather than direct viral infection of the CNS. Also, patient data from permeability imaging by MRI, in particular dynamic contrast-enhanced MRI, is emerging but still sparse. A pilot study investigated BBB integrity in seven survivors of severe COVID-19 who had been treated in an intensive care unit (ICU), comparing them to 17 healthy controls (Shi et al. 2023). Using a novel, non-invasive MRI technique which measures the permeability of the BBB to water without requiring external contrast agents, participants were assessed approximately three months after their discharge from ICU. They noticed that BBB permeability was significantly higher in the COVID-19 survivors compared with the control group. In contrast, there were no significant differences in whole-brain cerebral blood flow or brain volume between the two groups. These results provide preliminary evidence of chronic BBB breakdown following severe SARS-CoV-2 infection, suggesting that this impairment may be a primary contributor to Long COVID neurological symptoms, such as "brain fog" and cognitive decline. However, BBB breakdown is less likely to be cause of Long COVID neurological complications, for those non-hospitalized individuals initially only with mild symptoms, again requiring further investigation in this direction.

### SARS-CoV-2’s impact on pathological protein aggregation via different pathways

The molecular mechanisms of NDDs are complex and multifaceted, involving various cellular and molecular pathways beyond BBB dysfunction and immune dysregulation. Among the most accepted underlying mechanisms causing the major NDDs (i.e., AD and PD) are misfolding and abnormal aggregation of specific proteins, resulting in pathological protein aggregates related to neuronal damage (Wilson et al. 2023; Ochneva et al. 2022). Disease-specific proteins involved include Aβ and tau in AD and alpha-synuclein (α-syn) in PD (Tsoi et al. 2023).

The concept of protein seeding and spreading is often compared to prions, infectious proteins that cause conditions like Creutzfeldt-Jakob disease (Lee et al. 2010; Mudher et al. 2017; Costanzo and Zurzolo 2013). The seeding process is the initial event where a normal protein is transformed into a misfolded state (Soto and Pritzkow 2018). This misfolded protein may act as a template or seed, promoting the conversion of other physiologically folded proteins of the same species into misfolded forms. Once seeding has occurred, the misfolded proteins can spread from cell to cell, propagating the misfolding process. This spread can occur in several ways, including cell-to-cell transfer, through the extracellular space, or via neural networks (Soto 2012).

Extensive pathological, molecular, and genetic evidence in both animal models and humans has firmly established the significant role of abnormal forms of the protein α-syn in PD and other neurodegenerative disorders such as Lewy body dementia (LBD) or multiple system atrophy (MSA), all summarized as alpha-synucleinopathies (Calabresi et al. 2023; Ye et al. 2024). α-Syn can be detected in the CSF using seed amplification assays. CSF based α-syn seed amplification assays have been thoroughly tested and proven to be reliable showing consistent results in independent laboratories using clinically well-defined samples (Russo et al. 2021; Kang et al. 2019). Recently it was shown that a-syn seeds can be even used as blood-derived biomarkers for Synucleinopathies such as PD (Okuzumi et al. 2023). A model of was proposed correlating patho-anatomically spreading of a-syn with the progression of clinical symptoms in PD (Borghammer et al. 2021; Irwin et al. 2013). Together this suggests that the accumulation of α-syn preceding the onset of dopamine-related symptoms in some forms of PD might could serve as an early diagnostic marker in the future (Siderowf et al. 2023; Concha-Marambio et al. 2023).

NDD proteins are not infectious in the conventional sense, for instance, in AD Aβ peptides are produced from its full form (APP) and are involved in many physiological pathways of healthy neurons such as synaptogenesis, axonal growth, and synaptic plasticity (Müller et al. 2017). In a homeostatic context, these species can be degraded by Aβ proteases and/or engulfed by microglia, astrocytes and macrophages. Nevertheless, when accumulation occurs, Aβ particles exhibit prion-like behavior in their ability to induce misfolding and subsequent aggregation of normal proteins. The non-aggregated protein could be toxic, leading to the possibility that aggregation serves to isolate these harmful entities for detoxification. Therefore, it is proposed that aggregation would still be linked with the process as an epiphenomenon in this context. Nevertheless, this aggregation can also disrupt cell function by impairing cellular transport, damaging mitochondria, overwhelming the protein degradation machineries (proteasomes and lysosomes), and impairing synaptic transmission, all leading to cell death, causing the symptoms and progression of NDDs (Sweeney et al. 2017; Chung et al. 2018). Protein aggregates such as Aβ and tau induce structural brain changes and neuroinflammation, exacerbating further protein aggregation and neurodegeneration. This cyclical relationship between protein aggregation and neuroinflammation is evident in AD, where Aβ plaques and tau tangles actively engage microglia (Perry et al. 2010), amplifying neurotoxic effects and accelerating disease progression (Walker et al. 2019a). Also, inflammation is considered an early and pivotal event, influenced by genetic mutations and post-translational modifications that drive the formation of insoluble plaques or phosphorylated tau or a-syn deposits. Chronic neuroinflammation in the brain aggravates neuronal damage and induces BBB dysfunction (Takata et al. 2021). A compromised BBB allows more inflammatory cytokines, infiltrated immune cells, and possibly neurotoxic substances to enter the brain, further destabilizing neuronal environments and promoting protein aggregation (Takata et al. 2021; Yang et al. 2022).

**Fig. 3:**
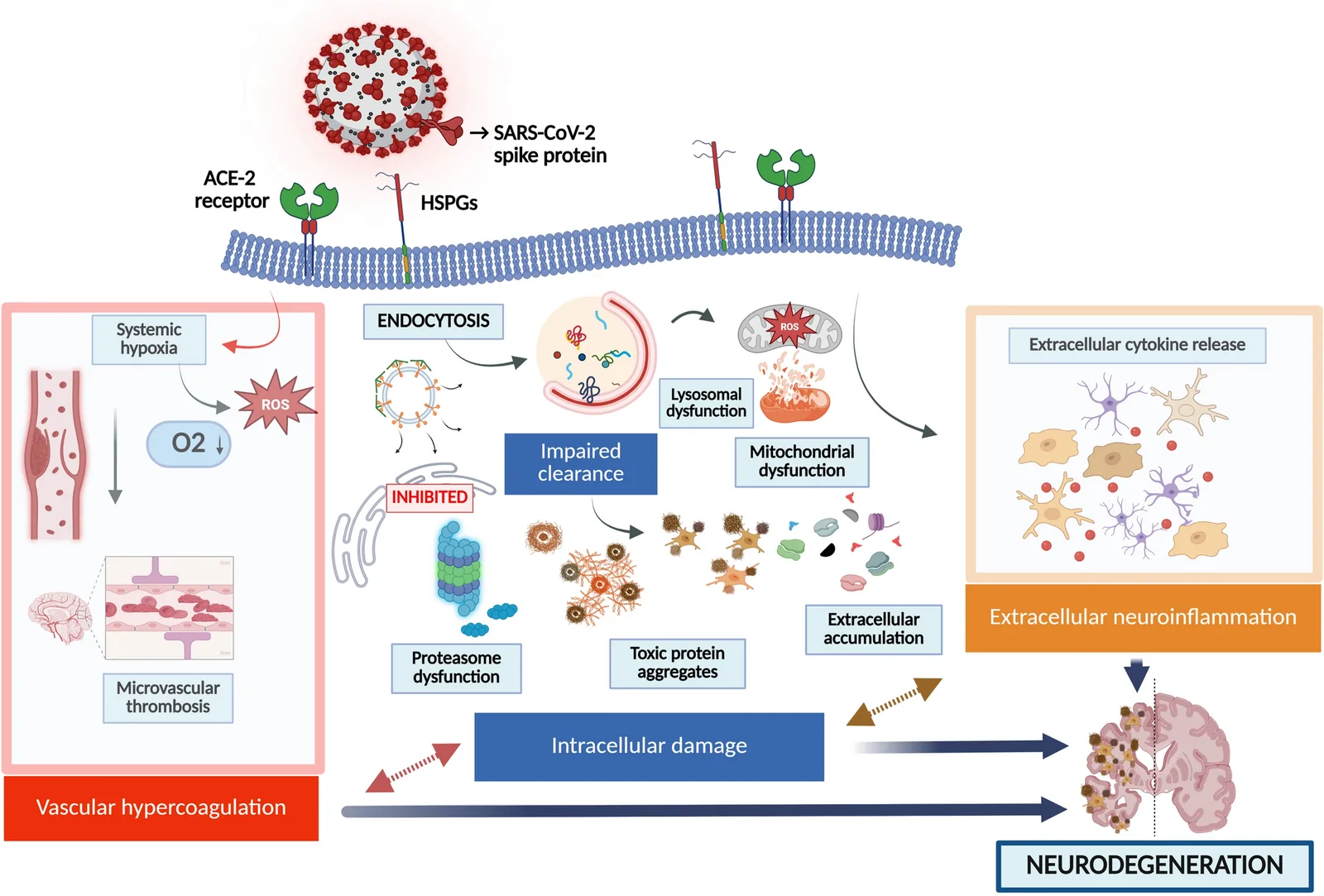
SARS-CoV-2’s impact on pathological protein aggregation via multiple mechanistic pathways. Schematic overview of the mechanisms by which SARS-CoV-2 might contribute to the seeding and spreading of pathological protein aggregates in the central nervous system. The spike protein (S) binds to ACE2 and HSPGs, facilitating the virus’ endocytosis. This viral entry pathway intersects key proteostatic systems, including the proteasome, lysosome, and autophagy networks. Viral interference disrupts endosomal trafficking and impairs protein clearance, leading to the intracellular and extracellular accumulation of misfolded and aggregated proteins. Mitochondrial dysfunction, triggered by S protein interactions or indirect oxidative stress, could further exacerbate protein misfolding via energy depletion, ROS production, and calcium imbalance. These events could activate the unfolded protein response (UPR) and promote chronic cellular stress. Ultimately, impaired clearance and inflammatory signaling (e.g., IL-1β from activated glial cells) might contribute to the propagation of toxic protein aggregates and neuronal injury, particularly in brain regions vulnerable to neurodegeneration. Hypercoagulation could result in a cascade of events, obstructing cerebral vessels, reducing blood flow to the brain, leading to neuronal injury or death and creating a state favorable to protein misfolding and aggregation. Furthermore, reduced blood flow and resultant hypoxia could increase oxidative stress in neuronal cells. ACE2, angiotensin-converting enzyme 2; HSPGs, heparan sulfate proteoglycans; ROS, reactive oxygen species.

SARS-CoV-2 could affect pathological protein aggregation process via multiple pathways (Fig. 3) that are shared with the onset and/or progression of NDDs. We will discuss different pathways in the following sections. The mechanisms discussed below represent a spectrum ranging from experimentally demonstrated cellular effects of SARS-CoV-2 and its S protein to biologically plausible hypotheses extrapolated from established pathways involved in neurodegeneration. While several of these mechanisms have been confirmed in vitro or in animal models, direct evidence demonstrating their contribution to pathological protein aggregation in the human brain in vivo remains limited (Nieuwland et al. 2026; Costa et al. 2025).

#### Via endocytosis

The lasting presence of the SARS-CoV-2 S protein within neurological tissues of both mouse models and human postmortem samples underscores its potential for long-term impacts (Rong et al. 2023). It has been proposed that SARS-CoV-2 or its S protein may facilitate pathological protein aggregation through several mechanisms and thereby contribute to neuropathological changes (Dolatshahi et al. 2021; Pacheco-Herrero et al. 2021; Lukiw et al. 2022). By binding to its receptors (i.e., ACE2) with intriguing cellular functions, SARS-CoV-2 or S protein could interfere with key cellular events, including cellular clearance mechanisms, cell signaling, and mitochondrial function, leading to the accelerated seeding and spreading of pathological protein aggregates in the brain (Villa et al. 2022). Once bound to ACE2 via its receptor-binding domain (RBD), the S protein is internalized by host cells through clathrin-mediated endocytosis (Bian and Li 2021). Cell surface heparan sulfate proteoglycans (HSPGs) act as co-receptors in this process (Fig. 3), facilitating S protein attachment and enhancing viral uptake (Clausen et al. 2020; Hudak et al. 2021). Beyond their role in viral entry, HSPGs are also critically involved in the uptake and intercellular propagation of amyloidogenic proteins such as Aβ, tau and α-syn, thereby representing a molecular link between SARS-CoV-2 infection and proteopathic spread (Letoha et al. 2019; Hudak et al. 2019). As clathrin-mediated endocytosis contributes to the clearance of extracellular monomeric misfolded proteins, overloading this pathway by SARS-CoV-2 or its S protein might impair internalization, leading to extracellular accumulation and the accelerated seeding of protein aggregates (Yu et al. 2014). Cells typically employ either organelles like lysosomes or protein complexes such as proteasomes to degrade internalized misfolded proteins or aggregates (Jackson and Hewitt 2016). In neurodegeneration, proteasomes primarily target soluble, ubiquitinated proteins and small oligomers, whereas lysosomes and the autophagy–lysosomal pathway degrade larger proteinaceous aggregates and damaged organelles, such as α-syn fibrils, tau tangles, and Aβ plaques (Ciechanover and Kwon 2015; Nixon and Rubinsztein 2024). These clearance systems are regulated by key modulators, including E3 ubiquitin ligases, autophagy-related proteins (e.g., ATG family), and molecular chaperones such as Hsp70, which guide misfolded proteins toward degradation (Johnston and Samant 2021).

Viral proteins, including the S protein, might directly interfere with this system by inhibiting proteasome function (Fig. 3) or indirectly by overwhelming the system with viral proteins (Gavilan et al. 2024). This interference could reduce the efficiency of protein degradation, leading to augmented protein aggregation. SARS-CoV-2 might induce lysosomal stress or damage, leading to the build-up of protein aggregates typically cleared by this pathway (Raaben et al. 2010). Endocytosis is also involved in the sorting and recycling of cellular components (Grant and Donaldson 2009). Should SARS-CoV-2 or S protein affect these functions, it could impair the cellular ability to degrade or recycle proteins correctly, contributing to accumulation of misfolded proteins and protein aggregation. The alteration of endocytic processes by SARS-CoV-2 could also induce cellular stress, potentially triggering the unfolded protein response and other stress responses, such as the production of reactive oxygen species (ROS) that could exacerbate neurodegeneration-associated protein misfolding (Gain et al. 2022; Abramov et al. 2020).

#### Via autophagy and lysosomal function

SARS-CoV-2 viral interference in endocytic pathways could also impact autophagic processes essential for clearing misfolded proteins and damaged organelles from cells. Viruses often manipulate autophagy to enhance their survival and replication (Fig. 3) (Kudchodkar and Levine 2009). For example, viral proteins can redirect autophagic machinery to avoid degradation or use autophagosomes for viral replication rather than degradation (Chen et al. 2023b; Chawla et al. 2022). This disruption can lead to the accumulation of cellular debris and protein aggregates, exacerbating conditions such as AD and PD. Importantly, autophagy is functionally dependent on lysosomal degradation: its final step requires fusion between autophagosomes and lysosomes (Yim and Mizushima 2020). If this fusion is impaired, or if lysosomal function is compromised, as it can occur during viral infection, autophagic flux stalls, and cargo accumulates (Chen et al. 2023a). Thus, lysosomal dysfunction acts as a bottleneck in the autophagy-lysosomal pathway, contributing to the pathological buildup of aggregation-prone proteins (Du et al. 2025).

#### Via interference with mitochondrial homeostasis

Direct interaction of S protein with mitochondrial proteins in neural and heart cells has been documented to induce changes in mitochondrial homeostasis and energy biodynamics in these high-energy-demanding tissues (Huynh et al. 2023; Clough et al. 2021; Pileggi et al. 2023; Shoraka et al. 2023; Bhowal et al. 2023). As mitochondria are the primary energy producers, their dysfunction leads to reduced ATP levels, which impairs various ATP-dependent cellular mechanisms, including an accumulation of misfolded proteins and an increasing the risk of aggregation (Bartman et al. 2024; Jishi and Qi 2022). Dysfunction in mitochondrial bioenergetics further compounds these effects, leading to energy deficits in neurons and contributing to the progression of NDDs (Johri and Beal 2012) (Fig. 3). Damaged mitochondria exacerbate the protein misfolding and aggregation through an increase in ROS production and alterations in calcium homeostasis (Zorov et al. 2014; Levy et al. 2019; Grune 2020; Klemmensen et al. 2024). The spike glycoprotein was shown to interfere with the ROS process (Pileggi et al. 2023). Disruption of redox homeostasis is tightly linked to abnormal protein dynamics and the combined dysfunction of the ubiquitin–proteasome system, autophagy, and lysosomal degradation, a triad of clearance mechanisms whose impairment is fundamental to the pathology of neurodegenerative disorders (Jellinger 2010; Ghavami et al. 2014). Interestingly, a genetic risk profile associated with increased susceptibility to mitochondrial damage has been described, suggesting a shared vulnerability axis between COVID-19 and NDDs (Magusali et al. 2021; Arena et al. 2023). Sustained mitochondrial impairment can lead to ATP depletion and oxidative stress, which may trigger the release of pro-apoptotic factors, initiating programmed cell death (D'Alessandro et al. 2025). Apoptosis itself might contribute to disease progression by releasing intracellular components, including misfolded or aggregation-prone proteins, into the extracellular environment, where they could seed further aggregation (Gorman 2008). For individuals with pre-existing neurodegenerative conditions such as AD or PD, mitochondrial stress induced by SARS-CoV-2 or its S protein could therefore accelerate disease progression by converging on energy failure, impaired clearance, and apoptosis-mediated propagation of protein aggregates (Elechi et al. 2025).

#### Via direct interaction with amyloidogenic proteins

Besides altering normal cellular physiology, the S protein can also directly interact with various amyloidogenic proteins, such as Aβ, α-syn or tau, leading to mitochondrial oxidative stress, apoptosis, and further neurodegeneration (Idrees and Kumar 2021; Rao et al. 2022; Mysiris et al. 2022) (Fig. 3). It has been proposed that the S protein containing sequence motifs with prion-like characteristics may interact with amyloidogenic proteins and potentially influence aggregation processes. Structural modelling studies and in vitro experiments support this possibility (Seneff et al. 2023; Zhao et al. 2022). Furthermore, recent experimental work demonstrated that SARS-CoV-2 and its S protein can induce extracellular Aβ aggregation in human retinal explants and retinal organoids, providing biological support for a direct link between viral proteins and pathological protein aggregation (Miller et al. 2025). This can lead to the accumulation of neurotoxic aggregates, further impairing neuronal function and exacerbating neurodegenerative conditions. The high binding affinity of SARS-CoV-2 proteins to α-syn can upregulate α-syn expression and accelerate its aggregation, potentially leading to the development of Lewy-body pathology, a hallmark of PD, in vitro (Semerdzhiev et al. 2022). Recent publications support the notion that COVID-19 may influence α-syn aggregation levels and play a part in neurodegenerative mechanisms (Blanco-Palmero et al. 2021). Together, these findings support the hypothesis that SARS-CoV-2 may facilitate proteopathic processes through direct interactions with amyloidogenic proteins, although the relative contribution of this mechanism to NDD progression remains to be clarified.

#### Via neuroinflammation

Further complicating the interaction of the S protein with neurological tissues is its ability to induce non-cell autonomous death of hippocampal neurons. This occurs through the expression of IL-1β from glial cells (Fig. 3), which is significantly upregulated following administration of the S1 protein. The presence of IL-1β is critical as it has been linked to neuronal cell death within the hippocampus (Oh et al. 2022). Additionally, the morphological changes in microglia, characterized by more circular cell bodies and shorter branches, suggest a disruption in their normal function, which is crucial for maintaining neuronal health. The presence of the virus or its components within the CNS activates microglia and astrocytes, leading to the release of inflammatory cytokines and chemokines (Almutairi et al. 2021). Chronic activation of these cells is linked to neurodegenerative processes and might enhance the aggregation of misfolded proteins or disrupt cellular mechanisms that clear these aggregates (Zhang et al. 2023). The persistent neuroinflammation within the brain further fuels ROS production, while the immune response might mistakenly target neuronal components, leading to an autoimmune response that might harm neurons and potentially promote further aggregation of pathologic proteins (Kempuraj et al. 2016).

#### Indirectly via hypercoagulation

SARS-CoV-2 or S protein does not have to enter the CNS to accelerate pathological protein aggregation. Severe COVID-19 has been associated with increased blood clotting. The S protein can contribute to this by affecting endothelial cells, causing endothelial dysfunction and inflammation, leading to a pro-thrombotic state and hypercoagulation (Perico et al. 2024). Blood clots and brain ischemia can result in a cascade of events, obstructing cerebral vessels, reducing blood flow to the brain, leading to neuronal injury or death and creating a state favorable to protein misfolding and aggregation (Fig. 3). Furthermore, reduced blood flow and resultant hypoxia could increase oxidative stress in neuronal cells (Wang et al. 2022b). As noted earlier, oxidative stress is a well-known factor that can induce protein misfolding. Chronic cerebral hypoperfusion due to ongoing vascular issues could exacerbate pre-existing neurodegenerative conditions or contribute to the development of new neurodegenerative pathology. The inflammatory response triggered by ischemia or the entry of immune cells through a compromised BBB can further exacerbate neuroinflammation, which has been shown to promote the seeding and spreading of pathological protein aggregates in NDDs. The long-term effects of increased clotting and its impact on the brain might contribute to a chronic state of reduced neurological function, promoting a milieu that accelerates neurodegenerative processes.

In short, the potential of SARS-CoV-2 or its S protein, particularly its amyloidogenic regions, to seed pathological protein aggregation presents a compelling link to neurodegenerative processes and systemic amyloidosis. The necessity to guarantee a further investigation becomes clear, paving the way for potential therapeutic innovations that address the direct and indirect long-term effects of SARS-CoV-2 on the CNS.

### The link between SARS-CoV-2 infection, inflammation and PD

It is quite straightforward to understand that the initiation of monogenic Parkinson’s disease (PD), especially in cases with early-onset mutations (i.e., PARK7/DJ-1) (Bonifati et al. 2003; Hague et al. 2003), is triggered by certain familial PD gene mutations. However, for idiopathic PD (iPD) patients, accounting for around 80–90% of all PD cases, it is still very challenging to pinpoint the precise triggering events of the disease onset, as almost 60% of dopaminergic neurons is already lost before the time of the first motor symptom (Greffard et al. 2006; Fearnley and Lees 1991; Dauer and Przedborski 2003) and the possible triggering events (Johnson et al. 2019) were postulated to exist decades before the onset of clinical symptoms (Ross et al. 2004). Among others, viral infection is thought to be one of the main triggering events for the onset of iPD cases. Notably, the incident rate of developing PD symptoms among people born during Spanish flu outbreak period was approximately doubled compared to before the Spanish flu pandemic period (Toovey et al. 2011; Vlajinac et al. 2013). In a Danish population-based study, the observed odds ratios raised by more than 70% and 90% for all PD occurring more than 10 years and 15 years after influenza infection, respectively (Cocoros et al. 2021). In line with those observations, a very recent large-scale epidemiological analysis based on > 400,000 participants has identified 45 significant pairs of viral-exposure-to-NDD (Levine et al. 2023). Interestingly, influenza with pneumonia was found significantly associated with five out of six studied NDDs, including PD. While epidemiological studies suggest an association between viral infection and increased PD risk, this relationship does not establish causality, highlighting the need for further investigation into the underlying pathogenic mechanisms (Lauritsen and Romero-Ramos 2023).

However, apparently, most individuals infected with influenza do not develop PD, indicating the existence of additional factors for the onset of PD. A potential contribution of the host immune system to neurodegeneration in PD has been postulated for a while (Mosley et al. 2012; Kannarkat et al. 2013). In the meantime, systemic immune dysregulations in both adaptive and innate immunity arms might be associated with PD occurrence (Fig. 1B), which has been nicely reviewed and summarized elsewhere (Floyd 1999; Perry et al. 2010; HeFeng and Balling 2013; Heppner et al. 2015; Tansey et al. 2022; Lauritsen and Romero-Ramos 2023). For instance, the increased levels of several cytokines in both serum and CSF (Dzamko 2023), especially TNF-α and IL-1β, have been reported by several original works (Brodacki et al. 2008; Reale et al. 2009) or meta-analyses (Qin et al. 2016). In line with this notion, two recent large-scale cohort studies have shown that systemic administration of anti-inflammatory drugs, is associated with significantly reduced PD incidence (Peter et al. 2018; Racette et al. 2018). Importantly, circulating T cells from iPD patients recognize α-syn peptide (Sulzer et al. 2017). The presence of α-syn-reactive T cells in the blood of PD patients suggests a potential immunological aspect to the pathogenesis, which may be especially relevant in the early or preclinical stages of the disease (Lindestam Arlehamn et al. 2020) further suggesting a role of T-cells in the pathogenesis of PD.

More specifically, a reduction of total CD4 T cells in PD was observed (Jiang et al. 2017). Furthermore, CD4 regulatory T cells (Treg) and Th17 cells in PD has been a topic of ongoing debate, with inconsistent findings from both animal models and clinical studies. Some studies have reported an increase in Th17 cells in PD patients (Sommer et al. 2018), while others have found no significant difference or even a decrease in Th17 cells compared to healthy controls (Kustrimovic et al. 2018), indicating the need of further investigations. A postmortem analysis shows that infiltration of CD8 T-cells has been found in brain tissues even before the α-syn aggregation and neuronal death (Galiano-Landeira et al. 2020). Very recently, using fresh blood, a study found that essentially, terminally-differentiated effector memory CD8 T cells (CD8 TEMRA) that are known to be highly cytotoxic (Wertheimer et al. 2014; Henson et al. 2012) were significantly enhanced in early-to-mid stage iPD accompanied with increased circulating cytotoxic molecule granzyme A (GZMA) but reduced CD8 FOXP3 + regulatory T cells (Capelle et al. 2023). The initial discovery was further validated in another sub-cohort, although only through cryopreserved samples. A similar observation has also been made by another team, demonstrating the feasibility to include cryopreserved samples and independently confirmed in another study (Wang et al. 2021c). Excitingly, a negative correlation between CD8 TEMRA and disease duration was found (Capelle et al. 2023), suggesting a contribution of CD8 TEMRA to PD pathogenesis. Mechanistically, the single-cell RNA-sequencing analysis demonstrated accelerated differentiation within CD8 compartments, enhanced cytotoxic pathways in CD8 TEMRA and effector memory CD8 T cells, while CD8 central memory and naïve cells were already more-active and transcriptionally-reprogrammed (Capelle et al. 2023). Coincidently, more abundant CD8 TEMRA cells have been also observed in several other NDDs across blood, CSF and CNS (Latour and McGavern 2026), possibly indicating a general pathogenic role of CD8 TEMRA cells in the onset and/or progress of NDDs. While the argument supports these intriguing findings, it has to be acknowledged that it is only based on a limited number of samples from small cohorts. Multi-centric or large-scale clinical studies are needed to consolidate the relevance and causality of these findings to the PD onset.

The emergence of COVID-19 has added a new dimension to the understanding of neuroinflammation in PD. Case reports of COVID-19-related parkinsonism (Merello et al. 2021), characterized by typical parkinsonian motor and non-motor symptoms arising during or shortly after infection, suggest that the virus may directly or indirectly impact neural tissues through mechanisms such as direct viral invasion, systemic inflammation, and hypoxic injury (Fig. 3). Furthermore, as discussed in an earlier section, increased plasma levels of TNF-α, IL-1β and IL-6 have been reported in individuals suffering from Long COVID for more than eight months (Schultheiß et al. 2022). In the meantime, many studies, especially a large-scale meta-analysis of > 150 different reports (Qu et al. 2023) and an independent middle-scale meta-analysis of > 20 works (Dzamko 2023), have shown that the same triad of cytokines have been found upregulated in both serum/plasma and CSF in PD patients compared with matched controls. The reproducibility of these cytokine findings across diverse cohorts and clinical studies ensures their robustness, particularly given the variability in disease stages and durations examined across studies. Although alterations in immune cell composition in Long COVID remain incompletely characterized, likely due to the so far lack of sufficiently deep immunophenotyping analyses, the convergence of pro-inflammatory cytokine signatures observed in PD and the systemic immune dysregulation associated with Long COVID suggests a potentially meaningful biological link. This immune response overlap has implications for understanding the potential triggers of neuroinflammation and neurodegeneration in PD and underscores the importance of considering viral infections in the broader context of NDD research (Cavallieri et al. 2022). However, despite the aforementioned case reports of Parkinson’s disease (PD) following SARS-CoV-2 infection and the observed overlap in specific circulating cytokine responses between PD and Long COVID, no convincing evidence has yet established a causal link between COVID-19 and an increased risk of PD, underscoring the need for further investigation. The lack of convincing causal evidence may be related to the multi-decade delay of PD development, whereas the COVID-19 pandemic occurred only recently.

### The link between SARS-CoV-2 infection, inflammation and AD

AD, more specifically, late-onset sporadic AD (LOAD), is the most prevalent type of dementia and the first cause of neurological disability in the elderly causing enormous social and economic burdens in modern societies (Diseases and Injuries 2024). It is characterized by progressive deficits in memory, and an increasing cognitive and behavioral impairment that ultimately leads to dementia (Breijyeh and Karaman 2020). The underlying pathomechanisms of LOAD are complex and not fully understood. However, its main pathological features include the presence of extracellular fibrillar aggregates of Aβ protein, which already occurs decades before the onset of the disease’s first symptoms, and intraneuronal aggregates of neurofibrillary tangles (NFTs) of phosphorylated tau protein, and neuronal cell death (Jack et al. 2013; Takahashi et al. 2017).

The most prominent AD genetic risk factor is the ε4 allele of the Apolipoprotein E (APOE) gene, present in approximately 65% of LOAD patients. ApoE is widely expressed in many body tissues, and recently it has been shown its interaction with the complement factor H. Factor H plays a central role in chemotaxis and inflammation by blocking complement component 3 (C3) opsonization and enhancing proinflammatory cascade of C3a. Notably, the ApoE4, the high-risk LOAD variant, binding to Factor H is impaired, leading to sustained inflammation and reduced Aβ aggregate clearing (Chernyaeva et al. 2023). It is also worth noting the genetic signatures of LOAD and their potential role in its pathomechanisms. Triggering receptor expressed on myeloid cells 2 (TREM2), an important AD gene associated with the microglia function, highlighting the genetic connection between the immune system and the risk to develop LOAD (Yeh et al. 2017).

Clinically, the presence of dementia could make patients with AD more susceptible to contracting COVID-19 and experiencing severe outcomes (Frontera et al. 2022), possibly linked to a combined effect of pre-existing pathological neuroinflammation added to the immune response to the infection (Fig. 1B). The presence of AD has been prevalent in hospitalized patients and deaths from COVID-19, even when corrected by age and other confounders (Ferini-Strambi and Salsone 2021). In the meantime, some studies have shown that individuals with APOE4 have an increased risk of severe responses to COVID-19. For example, SARS-CoV-2 infection may worsen neuropathology, especially in individuals with the APOE4 allele, which also increases the risk and severity of infection (Szabo et al. 2022; Bakker et al. 2022; Gaigher et al. 2022). This was consistent with in-vitro studies where APOE4 neurons are more susceptible to infection compared to APOE3 (Kuo et al. 2020; Wang et al. 2021a). APOE4 is also associated with a dysfunction of the BBB and cerebrovascular diseases, which are risks of severe COVID-19 outcomes and AD development (Montagne et al. 2020).

Interestingly, a two-sample Mendelian randomization analysis has shown a significant genetic correlation between the severity of COVID-19 and an increased risk of AD, indicating that genetic factors may also influence susceptibility to viral infections that could exacerbate neurodegenerative processes (Li et al. 2022). Some of the etiological factors of LOAD are common to COVID-19, which sustains the findings from recent studies (Rahman et al. 2021; Silva et al. 2022; Baazaoui and Iqbal 2022; Golzari-Sorkheh et al. 2023) on the cross-link between both diseases, suggesting that SARS-CoV-2 infection could accelerate neurodegenerative processes.

Numerous studies suggest that inflammatory processes are the most common mechanism involved in the development of LOAD. This complex interplay of factors contributes to the intricate pathology of AD (Twarowski and Herbet 2023) (Fig. 1B). Neuroinflammation, mainly mediated by astrocytes and microglia, is increasingly recognized as a contributor to LOAD pathogenesis. Microglial activation in the context of LOAD leads to the accumulation of neurotoxic proinflammatory astrocytes in the cortex and overexpressing the C3 complement factor (Liddelow et al. 2017). This process results in the secretion of multiple cytokines, secondarily feeding the positive feedback loop to enhance inflammation. In addition to neuroinflammation, the role of meningeal lymphatics has been associated with AD, and notably, a significant number of LOAD genetic risk factors are expressed by meningeal lymphatic endothelial cells, suggesting a drainage impairment in AD brains (Da Mesquita et al. 2021).

Although the direct association of LOAD and neuroinflammation is not fully understood, elevated systemic inflammation experienced during lifetime has been related to greater memory decline risk in a 20-year period (Walker et al. 2019b, 2018). Also, there is a significant correlation between infections happening up to 30 years before clinical diagnostic of dementia or death (Richmond-Rakerd et al. 2024). Interestingly, previous studies have also explored the link between infectious agents and AD, leading to the controversial “infectious AD hypothesis”. This hypothesis considers the infection with pathogens such as HSV a potential trigger for amyloid production in the brain (Wainberg et al. 2021; Sochocka et al. 2017). In fact, Aβ has antimicrobial peptide-like properties (Moir et al. 2018) and has been shown to inhibit the infectivity of influenza A virus by promoting viral aggregation (White et al. 2014), and similar protective effects have been described against HSV-1 (Bourgade et al. 2015). Increased Aβ production after infection may initially represent a host defense response to pathologic stress. Nevertheless, if sustained over time, excessive production or impaired clearance of Aβ may favor the accumulation of aggregation-prone species and ultimately contribute to plaque formation. The infectious AD hypothesis remains debatable, and infections are better considered potential environmental “second hits” that may interact with ageing, APOE genotype, immune dysregulation, impaired proteostasis, vascular dysfunction, or pre-existing Aβ and tau pathology.

Some interesting features shared between COVID-19 and AD, beyond APOE4, aging, and cardiovascular risk factors (Fig. 1A, B), are the state of systemic inflammation underlying both pathologies which share multiple markers. During the acute phase of SARS-CoV-2 infection, severe COVID-19 can be characterized by excessive systemic immune activation, frequently referred to as a “cytokine storm”, with increased circulating levels of proinflammatory cytokines such as IL-1β, IL-6, TNF-α, IL-12, IFN-γ and IL-17, which have been associated with disease severity and mortality (Lucas et al. 2020; Huang et al. 2020; Hadjadj et al. 2020). NLRP3 inflammasome is activated in response to SARS-CoV-2 infection and both IL-1β and IL-18 are downstream mediators of its activation (Rodrigues et al. 2021). Also as discussed in earlier sections, elevated levels of IL-1β, IL-6 and TNF-α have also been observed in individuals with long COVID eight months after infection (Schultheiß et al. 2022), although the majority initially only with mild infections, indicating that mechanisms beyond acute disease severity contribute to persistent symptoms (Al-Aly et al. 2022; Cai et al. 2024). IL-6 has also been reported to be elevated in the CSF of patients with neurological long COVID (Manganotti et al. 2021), supporting the possibility that systemic immune activation may be accompanied by the change of CNS inflammatory milieu in at least a subset of patients.

Several of these inflammatory mediators are also relevant to AD-related pathology. For instance, IL-1β can upregulate *APP* expression (Rogers et al. 1999), increase *BACE1* expression and Aβ production (Chami and Checler 2012), and promote tau phosphorylation through p38-MAPK signaling (Ghosh et al. 2013; Li et al. 2003), while the microglial NLRP3 inflammasome is activated by Aβ in the LOAD context (Ising et al. 2019; Baazaoui and Iqbal 2022).

Nevertheless, IL-1β, IL-6, TNF, IFN-related pathways and NLRP3 inflammasome activation are specific to neither COVID-19 nor AD but are components of common inflammatory responses across many conditions. Therefore, their presence in both diseases does not necessarily demonstrate a direct mechanistic chain from SARS-CoV-2 infection to AD pathology. Rather, these shared signatures may reflect convergent but partially independent inflammatory pathways that can arise from distinct insults. However, the aberrant activation of the immune system in response to SARS-CoV-2 infection could induce systemic inflammation leading to a cytokine storm, alterations of the BBB (Takata et al. 2021), causing synaptic defects and potentially enhancing oligomerization and aggregation of Aβ (Hong et al. 2016), due to a reduced microglial-mediated clearance, ultimately exacerbating the neurodegenerative pathology in AD patients or increasing the probability of developing AD (Golzari-Sorkheh et al. 2023; Villa et al. 2022).

Despite the hypotheses proposing a link between the inflammatory response triggered by SARS-CoV-2 and the progression of neurodegeneration potentially leading to an increased risk of AD, the long-term impact of COVID-19 on the brain remains uncertain and further research is required to explore the shared molecular signatures and pathways between COVID-19 and AD (Green et al. 2022; Frontera et al. 2022).

## Experimental models to probe those shared pathomechanisms

### Human iPSC-derived blood brain barrier (BBB)

Conventional animal models fail to faithfully reproduce human COVID-19 pathology and often rely on non-physiological, transgene-driven overexpression of the human SARS-CoV-2 receptor ACE2 (Bao et al. 2020; Sun et al. 2020) or on mouse-adapted viral strains to elicit disease signs (Dinnon et al. 2020). Although cell lines have studies of SARS-CoV-2 infection and drug testing (Ellinger et al. 2021), they do not mimic human cellular physiology and may lack key entry proteins such as ACE2, transmembrane protease serine 2 (TMPRSS2) (Hoffmann et al. 2020), or neuropilin-1 (NRP1) (Cantuti-Castelvetri et al. 2020b; Daly et al. 2020). These limitations highlight the need to develop or employ human-derived cellular models that more accurately reflect tissue-specific functions during SARS-CoV-2 infection.

**Fig. 4:**
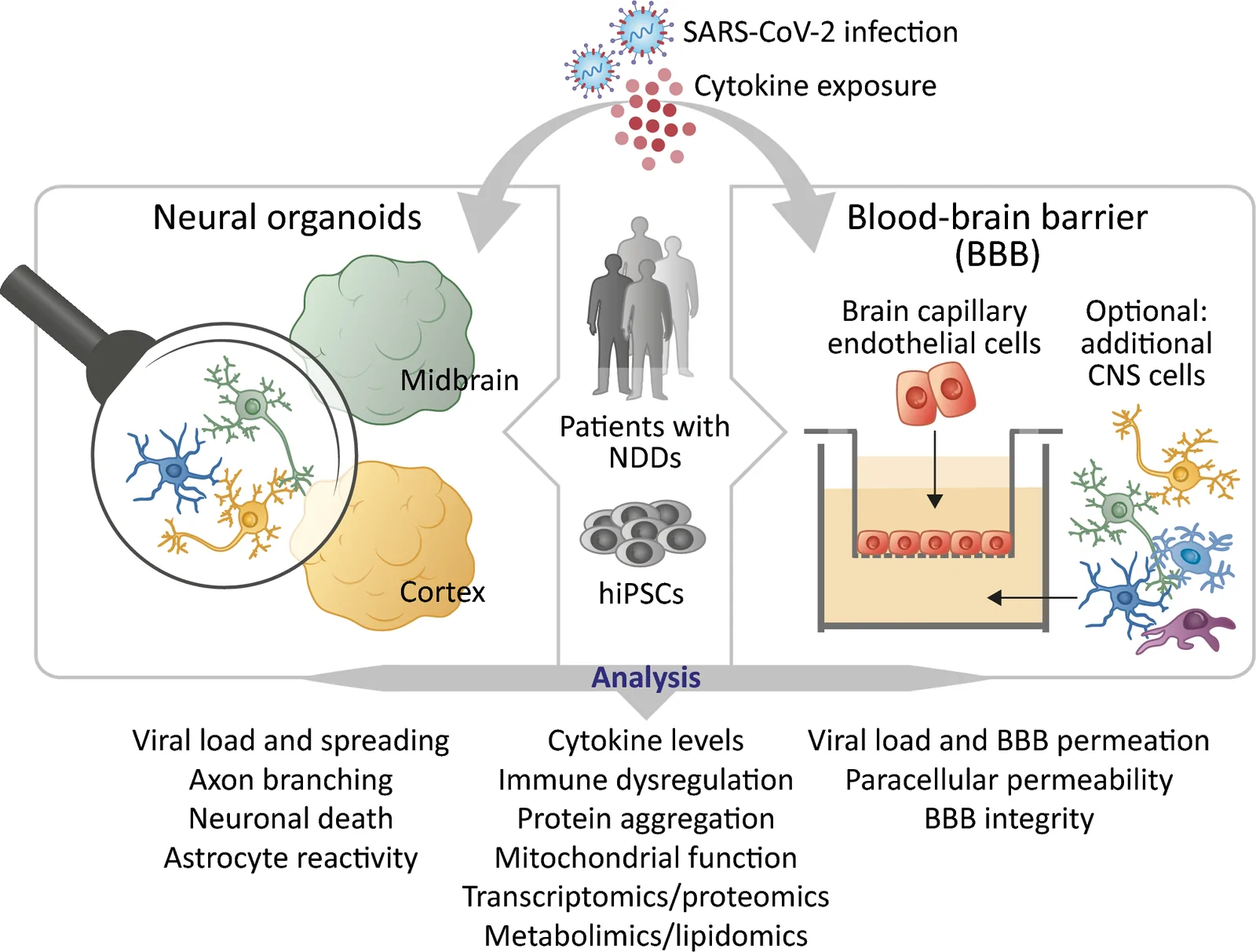
hiPSCs-derived experimental models employed to explore the link between SARS-CoV-2 infection and the onset of NDDs. *BBB* blood–brain barrier, *hiPSC* human induced pluripotent stem cell, *NDD* neurodegenerative disease

Human induced pluripotent stem cell (hiPSC)-based models provide a technology to investigate the susceptibility of different cell types to viral infection and the downstream molecular consequences. hiPSC-derived BBB models (Fig. 4) have been successfully used to study infection by bacteria, viruses (including SARS-CoV-2 and Zika virus), and fungal toxins (Alimonti et al. 2018; Kim et al. 2017; Martins Gomes et al. 2019; Patel et al. 2019; Kim and Schubert-Unkmeir 2019; Krasemann et al. 2022). hiPSC-derived organoids have been used to model SARS-CoV-2 infection in several organs, including the vasculature (Monteil et al. 2020) and brain (Ramani et al. 2020). These experiments have shown that SARS-CoV-2 can infect and replicate within cells across multiple organs, leading to changes in expression in genes linked to inflammatory responses and altered cellular functions.

One way to generate tight vasculature models from hiPSC-derived brain capillary endothelial cells (hiPSC-BCECs) was suggested in 2012 (Lippmann et al. 2012) and refined since (Lippmann et al. 2014; Appelt-Menzel et al. 2017). hiPSC-BCECs exhibit a transcriptome which is highly comparable to that of freshly isolated brain vessels from human brain biopsies (Krasemann et al. 2022). This model has successfully been combined with other cell types of the neurovascular unit (NVU), i.e., pericytes, astrocytes, and neuronal cells (Appelt-Menzel et al. 2017). This hiPSC-BCEC model provides a robust and reproducible tool to study SARS-CoV-2 infection and BBB-related molecular consequences (Krasemann et al. 2022). Also, cell-intrinsic genetic disease-associated risk factors, e.g., APOE4/4 haplotypes, can be studied in such a scenario (Jung et al. 2023; Haferkamp et al. 2023; Hartmann et al. 2026). As a result of the infection, hiPSC-BCECs show SARS-CoV-2-induced pathophysiological changes at a cellular and molecular level, in particular a strong type I IFN response. Importantly, by design of the assay, this upregulation of IFN signaling as a result of SARS-CoV-2 infection, is independent from any immune cell contribution.

### Human iPSC-derived neurodegenerative disease models: from 2D culture to 3D organoids

In the last two decades, research on NDDs has experienced a wave of significant breakthroughs thanks to the advent of iPSCs-derived in vitro neuronal models. Since then, multiple iPSC-derived models have been developed to study the mechanisms underlying AD, both familial and LOAD (Zhao et al. 2020; Penney et al. 2020; Shi et al. 2012; Wang et al. 2018; Espuny-Camacho et al. 2017).

Researchers have demonstrated that these hiPSC-derived models can recapitulate the pathophysiology of AD in vitro. Numerous papers have already been published using hiPSC-derived neurons with familial AD (fAD)-related mutations showing a high proportion of Aβ peptides and hyperphosphorylated tau protein, along with electrophysiological hyperactivity and autophagy, defective endocytosis and mitophagy (Ochalek et al. 2017; Kondo et al. 2013; Chang et al. 2019; Wezyk et al. 2018; Li et al. 2018; Lee et al. 2014; Martin-Maestro et al. 2017). Many of these features are also present in hiPSC-derived neurons generated from sporadic AD (sAD) patients, although the phenotypes are more diverse due to the genetic heterogeneity of sAD (Ochalek et al. 2017; Lavaud et al. 1990; Israel et al. 2012; Birnbaum et al. 2018). For example, hiPSC-derived neurons carrying the APOE4 risk factor, the major genetic risk factor associated with sAD, show increased Aβ production, elevated tau phosphorylation and alterations in endosomal and autophagy functions compared with neurons carrying the APOE3 allele (Lin et al. 2018; Wang et al. 2018; Duan et al. 2014; Wadhwani et al. 2019). Regarding hiPSC-derived astrocytes generated from either fAD-linked (PSEN1^M146L^) or sAD patients (APOE4 carrier), display a similar phenotype, characterized by reduced morphological complexity and mislocalized marker proteins (Wadhwani et al. 2019). These astrocytes can affect the survival and function of co-cultured neurons, showing reduced Aβ uptake, increased inflammatory signaling and altered cholesterol metabolism (Wadhwani et al. 2019; Oksanen et al. 2017; Zhao et al. 2017; Lin et al. 2018; de Leeuw et al. 2022). Regarding hiPSC-derived microglia cultures, these models reveal alterations in phagocytosis and gene expression in response to AD-associated mutations (Lin et al. 2018; Abud et al. 2017; Xu et al. 2019). Specifically, APOE4 microglia cells exhibit a pro-inflammatory phenotype and impaired Aβ clearance (Lin et al. 2018), while also exacerbates microglia dysfunction by driving them into a state characterized by lipid droplet accumulation (Haney et al. 2024). Microglia carrying TREM2 mutations show decreased viability and impaired phagocytic ability (Claes et al. 2019; Garcia-Reitboeck et al. 2018).

The development of co-culture systems and in particular of 3D organoid models and from hiPSCs (Fig. 4) revolutionized the possibilities to study NDDs. Organoids recapitulate key structural and functional features of human organs. Their multicellular architecture and physiological relevance enable disease modeling, drug discovery, and toxicity testing (Wittich et al. 2026). They more accurately reproduce brain architecture and disease characteristics compared to conventional 2D cultures. Indeed, they have allowed researchers to observe the formation of Aβ plaques and tau tangles, key features of AD. Another difference to 2D cultures is that neural organoids secrete their extracellular matrix (enriched in proteoglycans, hyaluronic acids, matrix metalloproteases, among others), which influence the formation of amyloid plaques (Bejoy et al. 2018; Yan et al. 2018; Rahman and Lendel 2021). Furthermore, researchers have observed proteomic changes in organoids similar to postmortem human tissues, including proteins involved in axonal damage, oxidative stress and neuroinflammation, thus demonstrating their physiological relevance (Chen et al. 2018).

Studies using organoids with fAD mutations (in APP and PSEN1) or sAD (APOE4 carriers) have consistently shown increased amyloid plaques and tau phosphorylation (Choi et al. 2014; Raja et al. 2016; Seo et al. 2017; Kwak et al. 2020; Kuehner et al. 2021; Zhao et al. 2020; Hernandez et al. 2022). Some models also exhibit mitochondrial dysfunction, oxidative stress, and altered synaptic density (Chen et al. 2018; Zhao et al. 2020; Park et al. 2021), while others display asynchronous calcium transients and enhanced neuronal hyperactivity (Yin and VanDongen 2021; Ghatak et al. 2019). In addition to genetic factors associated with AD, environmental factors contributing to LOAD are being studied with neural organoids. These include treatments that induce AD-like pathology (Pavoni et al. 2018; Chen et al. 2021b) and exploration of the “Antimicrobial Protection Hypothesis” (Moir et al. 2018), which links viral infections to amyloid production and neurodegeneration (Spitzer et al. 2016). Along this line, other studies have shown that infection of neural organoids with HSV-1 (Cairns et al. 2020; Kong et al. 2024) or Zika virus (Lee et al. 2022) triggers AD-like pathology, including amyloid plaques, gliosis and neuroinflammation. Despite all these advantages, organoids also show limitations when compared to native tissues. Most organoid models lack vascularization and related sheer stress due to blood flow, (peripheral) immune cell interactions, microbiomes, and full cellular diversity and functionality (Chang et al. 2021; Ouchi and Koike 2023). Additionally, they lack consistent cellular composition and exhibit considerable variability in formation, morphology, and functionality, which often hampers their usage in disease modeling due to variability in self-organization and cell fate decisions (Hofer and Lutolf 2021).

### Infection in human iPSC-derived neural organoids

The SARS-CoV-2 virus has been found in neurons across various regions of human postmortem tissue samples (Matschke et al. 2020; Meinhardt et al. 2021; Yang et al. 2021; Paniz-Mondolfi et al. 2020), together with signs of astrogliosis (Matschke et al. 2020; Al-Dalahmah et al. 2020; Duarte-Neto et al. 2020; Kanberg et al. 2020; Reichard et al. 2020; Younger 2021), microgliosis (Matschke et al. 2020; Duarte-Neto et al. 2020; Younger 2023), axonal degeneration (Paniz-Mondolfi et al. 2020; Kanberg et al. 2020; Reichard et al. 2020; Younger 2021) and cell death (Yang et al. 2021; Younger 2021). Nevertheless, the study of acute SARS-CoV-2 infection is a difficult task due to the impossibility of obtaining brain biopsies from individuals at the time of the acute infection. The human brain uses its own mechanisms to clear the infection, so the collection of tissue long after this process could be disconnected from the actual infectious process. Additionally, many longitudinal studies lack information on vaccination status, co-infections, and viral strain. For these reasons, organoid cultures have been used in recent years to study viral infections in a more manageable and controlled environment (Depla et al. 2022). Neural organoids (Fig. 4) are 3D long-term in vitro cultures that recapitulate the basic cytoarchitecture and function of the human brain tissue. Neural organoids can model several regions of the brain such as forebrain, midbrain or hindbrain; more specific regions such as cortex, thalamus, or spinal cord; and even specialized areas such as choroid plexus (ChP) or retina (Kelley and Pasca 2022).

Of all the known cell types that can be infected, ChP cells have been described as the brain cell type exhibiting the highest susceptibility to SARS-CoV-2 infection using neural organoids (Jacob et al. 2020; Pellegrini et al. 2020). ChP cells are specialized epithelial cells that act as a selective gateway for the pathogens to the brain, and are part of the blood-CSF brain barrier (BCSFB). Mature ChP cells have high expression of the receptor ACE2 and the co-entry factor TMPRSS2, which has not been clearly observed in astrocytes (Andrews et al. 2022). Although ACE2 expression in neural cells remains controversial (Pellegrini et al. 2020; Song et al. 2021), other entry receptors have been proposed to serve as entry factors for SARS-CoV-2 virus, such as DPP4 (CD26) and BSG (CD147) for astrocytes (Andrews et al. 2022), and NRP1 for neurons (Cantuti-Castelvetri et al. 2020a) and astrocytes (Kong et al. 2022).

SARS-CoV-2 can infect ChP cells (Jacob et al. 2020; Pellegrini et al. 2020). Productive SARS-CoV-2 infection disrupts ChP epithelium, as evidenced in organoids by the delineation of the tight-junction protein CLDN5, a dramatic decrease in internal CSF-like fluid volume and an increase in APOJ protein release to the media (Pellegrini et al. 2020). ChP cells can also contribute to the immune response by secreting cytokines to the CSF. Upregulation of inflammatory cytokines such as CCL7, IL-32, CCL2 (MCP1), IL-18, and IL-8 is detected 72 h post-infection (hpi), together with elevated levels of phospho-p38 in both infected and nearby uninfected cells, suggesting a non-cell-autonomous effect happening due to the infection (Jacob et al. 2020).

Clinical SARS-CoV-2 isolate is known to infect neurons of the human brain (Song et al. 2021). Syncytia formation by cell–cell fusion has been proposed as the mechanism for viral propagation in neural cells (Wang et al. 2021a; Jacob et al. 2020). Elevated levels of neural cell death following SARS-CoV-2 infection have been documented in infected neural organoids, showing a positive correlation with the density of infected cells (Song et al. 2021; Samudyata et al. 2022; Ramani et al. 2020). Additionally, there is evidence of mis-localization of axonal tau protein to the soma and early tau phosphorylation at T231, suggesting potential neuronal stress related to neurodegeneration as early as 48 hpi (Ramani et al. 2020). Metabolic alterations associated with mitochondrial metabolism and responses to hyperoxia have been observed in infected neurons, while neighboring neurons exhibit cellular responses indicative of a local hypoxic environment (Song et al. 2021). Dopaminergic neurons have been identified as vulnerable to SARS-CoV-2 infection, resulting in the manifestation of a senescent phenotype characterized by heightened levels of beta-galactosidase, lysosomal accumulation, mitochondrial dysfunction, and increased ROS (Yang et al. 2024).

SARS-CoV-2 was also reported to infect and replicate in astrocytes (Andrews et al. 2022). This infection leads to an increased abundance of CD14, interferon induced transmembrane protein 3 (IFITM3), and TREM2, accompanied by the upregulation of inflammatory genes involved in type I IFN and IFN-γ signaling, as well as TNF production. Interestingly, TREM2 gene has been associated with genetic AD risk (Guerreiro et al. 2013) and it works as the Aβ receptor in microglia (Zhao et al. 2018) and correlates with tau phosphorylation levels in CSF (Heslegrave et al. 2016). Additionally, infected astrocytes display signs of reactive astrogliosis, as evidenced by elevated levels of GFAP and STAT3, along with enhanced phagocytic capacity indicated by increased expression of multiple epidermal growth factor-like domains 8 (MEGF8) and ATP-binding cassette subfamily A member 1 (ABCA1) (Samudyata et al. 2022).

SARS-CoV-2 infection affects microglia, leading to increased expression of activation markers such as TREM2, CD4, secreted phosphoprotein 1 (SPP1), and integrin subunit Alpha X (ITGAX) in infected cortical tissue in organotypic culture (Andrews et al. 2022). This activation persists for up to seven weeks post infection in subcortical regions in mouse models and is observed in both cortex and subcortex in human postmortem samples (Fernandez-Castaneda et al. 2022). Transcriptomic analysis reveals a shift in the microglial population towards increased expression of IFN-stimulated genes and the NFKB1 gene 72 hpi. Immunofluorescent staining shows microglia engulfing postsynaptic termini, accompanied by significant structural changes towards a less ramified morphology (Samudyata et al. 2022). Microglia in infected mice exhibit heightened production of cytokines and chemokines seven days post infection, increased cytotoxicity and apoptosis, antigen processing and presentation, and overall immune responses, which are known to impair hippocampal neurogenesis and are reminiscent of processes seen in AD and aging (Fernandez-Castaneda et al. 2022).

The extent of cell infection varies in scientific literature based on factors such as the multiplicity of infection (MOI) utilized for SARS-CoV-2 exposure, organoid age, type and genotype. For instance, mature astrocytes are absent in young organoids, and ChP cells and microglia are absent in pure cortical organoids. In addition, most neural organoid systems remain closer to fetal/neurodevelopmental tissue than to the adult brain and do not fully reproduce long-range circuitry, cortical layering, gyrification, vascular perfusion or systemic immune inputs (Bhaduri et al. 2020; Qian et al. 2019; Mulder et al. 2023). This is particularly relevant to SARS-CoV-2 studies, because the absence of vascular and immune components can limit modelling of BBB/BCSFB-mediated viral entry, endothelial responses, circulating cytokines and immune-cell recruitment (Birtele et al. 2025). Moreover, diffusion limitations can induce hypoxic/necrotic cores and culture-related stress pathways, which may confound interpretation of viral cytotoxicity, metabolic changes and inflammatory signatures (Qian et al. 2019; Bhaduri et al. 2020). Thus, neural organoids represent a valuable and experimentally controlled, but still model-dependent platform to study viral effects in human neural tissue, especially when interpreted together with postmortem data, animal models and more complex vascularized or immune-competent organoid systems.

## Computational knowledge graphs (KG) facilitate mechanistic understanding

Biological knowledge is inherently complex, distributed across many scales, and fragmented across thousands of studies, databases, and experimental systems. Knowledge graphs (KGs) provide a formal framework to integrate heterogeneous biological data layers, such as genes, proteins, pathways, phenotypes, and diseases, into a unified, machine-readable representation that preserves biological relationships. KGs are particularly valuable for studying disease comorbidities, as they enable the systematic comparison of molecular mechanisms across seemingly distinct conditions. They offer a computable repository of knowledge that can be queried and analyzed using various computational tools. In the domain of biological research, KGs are essential for integrating vast amounts of heterogeneous biological data in a harmonized way and mapping the complex associations between diverse entities such as genes, proteins, drugs, diseases, and different biological processes to uncover hidden patterns (Nicholson and Greene 2020). When combined with artificial intelligence (AI) tools, including advanced ML/deep learning (DL) architectures and large-language models (LLMs), they play a crucial role in enhancing our understanding of disease mechanisms and progression. They aid in identifying key biomarkers or pathways and offer new perspectives on potential therapeutic interventions (Fernandez-Torras et al. 2022; Santos et al. 2022; Zheng et al. 2021). Their utility can be extended to comprehend the complex interplay between COVID-19 and NDDs.

Prominent biomedical KGs, such as PrimeKG (Chandak et al. 2023) and HetioNet (Himmelstein et al. 2017), provide a systems-level view of biological relationships by mapping extensive networks of gene-disease associations and diverse biological interactions. However, their generalized structure often lacks the specificity required to accurately model the intricate pathophysiological mechanisms underlying complex diseases. Manually curated, disease-specific KGs and disease maps overcome this limitation by providing more focused insights into the molecular and cellular processes driving particular conditions. In the context of NDDs and COVID-19, NeuroMMSig AD&PD KGs (Domingo-Fernandez et al. 2017) and COVID-19 KG (Domingo-Fernandez et al. 2021), both represented in Biological Expression Language (BEL) format, provide literature-derived cause-effect relationships specific to these conditions. Similarly, disease maps including AlzPathway (Mizuno et al. 2012), Parkinson’s Disease Map (Fujita et al. 2014), and COVID-19 Disease Map (Ostaszewski et al. 2020, 2021), encoded in Systems Biology Markup Language (SBML), enable comprehensive modelling and simulation of biological processes. These curated resources map out key signaling pathways, metabolic processes, and gene regulatory networks that are central to disease progression, with evidence of their relationships to pathophysiological causes and outcomes. They capture dysregulated pathways and mechanisms, such as the accumulation of tau and Aβ in AD or the role of α-syn aggregation and mitochondrial dysfunction in PD, while also accounting for multifactorial triggers like oxidative stress and neuroinflammation. COVID-19 Disease Map and COVID-19 KG illustrate viral-host interactions, immune responses, and the systemic impacts of SARS-CoV-2 infection.

The utility of these resources in illuminating the COVID-neurodegeneration interface has been shown in multiple studies. For instance, the NeuroMMSig AD&PD KGs and the COVID-19 KG, alongside additional curated sources including KEGG AD and PD pathways, IntAct, and BioGRID, were intersected to systematically identify shared molecular mechanisms across all three disease etiologies (Linden et al. 2021). This analysis revealed convergent roles for innate immune sensing, inflammasome activation and downstream cytokine secretion, and apoptotic regulation across COVID-19, AD, and PD. Notably, IL-6 and IL-10, secreted downstream of inflammasome activation, were confirmed as predictive markers of COVID-19 severity, providing experimental grounding for the intersection-derived hypothesis. From this analysis, tyrosine kinase 2 (TYK2) emerged as a particularly compelling shared node, implicated in the amyloid cascade and α-syn-induced neuroinflammation, patented as a drug target in AD, and genetically associated with COVID-19 severity through Genome-Wide Association Study (GWAS) studies. Downstream experimental validation identified Regorafenib and Sorafenib, both are reported to be non-selective Janus kinase (JAK)/TYK2 inhibitors, as exhibiting antiviral activity against SARS-CoV-2 in cellular assays, providing proof-of-concept that mechanistic intersection of disease-specific KGs can generate experimentally actionable therapeutic hypotheses at the interface of COVID-19 and neurodegeneration. Similarly, in another study (Babaiha et al. 2025), a multi-scale graph analysis integrating KGs built from NeuroMMSig AD&PD KGs, COVID-19 KG, PrimeKG, DrugBank, KEGG, DisGeNET, OpenTargets, and natural language processing (NLP)-derived text mining sources was used to investigate the molecular and phenotypic links between COVID-19 and NDDs through path analysis, phenotype coverage, and mapping of cellular and genetic factors. Across all graph sources, immune response genes and inflammatory markers, including TLR7, VEGFA, APOE, and multiple cytokines (TNF, IL-2, IL-21), consistently emerged as central shared nodes, suggesting a common pathogenic basis, and GWAS-based cross-validation further reinforced the genetic overlap between COVID-19 susceptibility and neurodegenerative conditions. These findings are further corroborated by another work (Zhou et al. 2021) where the authors applied a network proximity framework to the SARS-CoV-2 virus-host interactome alongside neurological disease gene sets, confirming mechanistic overlap between COVID-19 and AD centered on neuroinflammation and brain microvascular injury, validated across transcriptomics, proteomics, and CSF biomarker data from COVID-19 patients. Independent transcriptomic intersection analyses similarly identified neuroinflammation and immune response pathways as differentially expressed gene signatures shared across COVID-19 and AD datasets (Fan et al. 2024), confirming that the mechanistic overlaps surfaced by these KG-based analyses reflect genuine biological signal rather than graph-structural artefact.

KG approaches are valuable for exploring mechanistic insights but have inherent limitations. Automated literature-mined KGs often capture statistical co-occurrence rather than causality, while association-based databases similarly lack clear directional relationships (Jiang et al. 2022). Manually curated resources such as NeuroMMSig and the COVID-19 KG address this by encoding causal relationships, but their development is slow, resource-intensive and subject to curator bias. Across all approaches, KG quality remains constrained by source data, leading to underrepresentation of understudied entities and the risk of reinforcing existing knowledge biases rather than uncovering new mechanisms (Nicholson and Greene 2020; Issa et al. 2021). Thus, KGs are best viewed as hypothesis-generation tools requiring experimental validation. A major gap remains in comorbidity graph modelling that integrates COVID-19 with NDDs in a unified framework. Developing such causally annotated, comorbidity-focused resources could provide deeper insight into how COVID-19 may influence the progression of AD and PD, with important implications for therapy and long-term care.

## Conclusions

Our systematic examination of the interplay between SARS-CoV-2 infection and neurodegenerative mechanisms reveals a complex, multifaceted relationship that underscores the virus’s capability to exacerbate or potentially initiate processes leading to neurodegeneration. Through its mechanisms of neural invasion, interaction with the blood–brain barrier, contribution to systemic inflammation and facilitation of protein aggregation, SARS-CoV-2 has been identified as a significant factor in neurological complications, both directly and indirectly. Substantial differences in clinical severity observed between different SARS-CoV-2 variants, e.g., between Omicron and earlier variants, shall prompt us to investigate the underlying SARS-CoV-2 variant-specific comorbidity mechanisms that link to the onset of NDDs. On the other hand, one also need to bear in mind the potential risk reduction perspective on the development of NDDs following SARS-CoV-2 vaccination if the effect is similar to that of herpes zoster vaccines (Eyting et al. 2025). These intertangled layers highlight the urgent need for deeper research and development of targeted therapeutic interventions, e.g., targeting IL-6 and JAK-STAT pathways (Gao et al. 2025), to address these neurological impacts, underscoring the importance of understanding the involved key molecular pathways.

NDDs, such as AD and PD, are influenced by both genetic and environmental factors (Chin-Chan et al. 2015; Ohnmacht et al. 2020; Dorsey and Bloem 2024). Specific gene mutations have been identified that increase the risk of these diseases. For instance, mutations in the APP, PSEN1, and PSEN2 genes are associated with early onset AD (Lanoiselee et al. 2017) while mutations in the LRRK2, PARKIN, PINK1, SNCA are linked to PD (Nuytemans et al. 2010). However, neurodegeneration is rarely caused by single gene mutation; the majority of the so-called idiopathic cases are believed to result from a combination of complex genetic modifiers, environmental influences and lifestyle factors. In the meantime, individuals with certain genetic mutations [e.g., the genes in the type 1 IFN pathway (Zhang et al. 2020) among others] that are not yet part of well-established PD or AD familial genes may have a higher vulnerability to environmental toxins and severe viral infections, which could in turn potentially hasten the progression of neurodegenerative disorders.

The complex interplay between genetic predispositions, infectious diseases and environmental exposures can significantly impact the onset and progression of NDDs, suggesting avenues for future research that could lead to novel preventive measures or treatment options. The works discussed herein emphasize the critical importance of advancing our knowledge in this area to combat the long-term neurological consequences of COVID-19 better and protect those most vulnerable to its long-reaching impacts.

## Data Availability

No original datasets were generated or analysed in the current review.
